# Characterization of the ZCTs, a subgroup of Cys2-His2 zinc finger transcription factors regulating alkaloid biosynthesis in *Catharanthus roseus*

**DOI:** 10.1007/s00299-024-03295-8

**Published:** 2024-08-08

**Authors:** Krystyna K. F. Traverse, Samuel Breselge, Juliet G. Trautman, Amanda Dee, Jie Wang, Kevin L. Childs, Carolyn W. T. Lee-Parsons

**Affiliations:** 1 Department of Chemical Engineering, Northeastern University, Boston, MA 02115 USA; 2 Department of Biology, Northeastern University, Boston, MA USA; 3 Department of Bioengineering, Northeastern University, Boston, MA USA; 4 Department of Plant Biology, Michigan State University, East Lansing, MI USA; 5 Department of Chemistry and Chemical Biology, Northeastern University, Boston, MA USA

**Keywords:** Cys2-His2 zinc finger transcription factor, *Catharanthus roseus*, ZCT, Terpenoid indole alkaloid, Transcriptional repressor, Jasmonate, MYC2a, EAR-motif

## Abstract

**Key Message:**

The *C. roseus ZCTs* are jasmonate-responsive, can be induced by CrMYC2a, and can act as significant regulators of the terpenoid indole alkaloid pathway when highly expressed.

**Abstract:**

*Catharanthus roseus* is the sole known producer of the anti-cancer terpenoid indole alkaloids (TIAs), vinblastine and vincristine. While the enzymatic steps of the pathway have been elucidated, an understanding of its regulation is still emerging. The present study characterizes an important subgroup of Cys2-His2 zinc finger transcription factors known as **Z**inc finger ***Catharanthus*** **T**ranscription factors (ZCTs)*.* We identified three new ZCT members (named ZCT4, ZCT5, and ZCT6) that clustered with the putative repressors of the TIA pathway, ZCT1, ZCT2, and ZCT3. We characterized the role of these six ZCTs as potential redundant regulators of the TIA pathway, and their tissue-specific and jasmonate-responsive expression. These ZCTs share high sequence conservation in their two Cys2-His2 zinc finger domains but differ in the spacer length and sequence between these zinc fingers. The transient overexpression of *ZCTs* in seedlings significantly repressed the promoters of the terpenoid (*pLAMT*) and condensation branch (*pSTR1*) of the TIA pathway, consistent with that previously reported for ZCT1, ZCT2, and ZCT3. In addition, ZCTs significantly repressed and indirectly activated several promoters of the vindoline pathway (not previously studied). The *ZCTs* differed in their tissue-specific expression but similarly increased with jasmonate in a dosage-dependent manner (except for *ZCT5*). We showed significant activation of the *pZCT1* and *pZCT3* promoters by the de-repressed CrMYC2a, suggesting that the jasmonate-responsive expression of the *ZCTs* can be mediated by CrMYC2a. In summary, the *C. roseus ZCTs* are jasmonate-responsive, can be induced by CrMYC2a, and can act as significant regulators of the TIA pathway when highly expressed.

**Supplementary Information:**

The online version contains supplementary material available at 10.1007/s00299-024-03295-8.

## Introduction

**Fig. 1:**
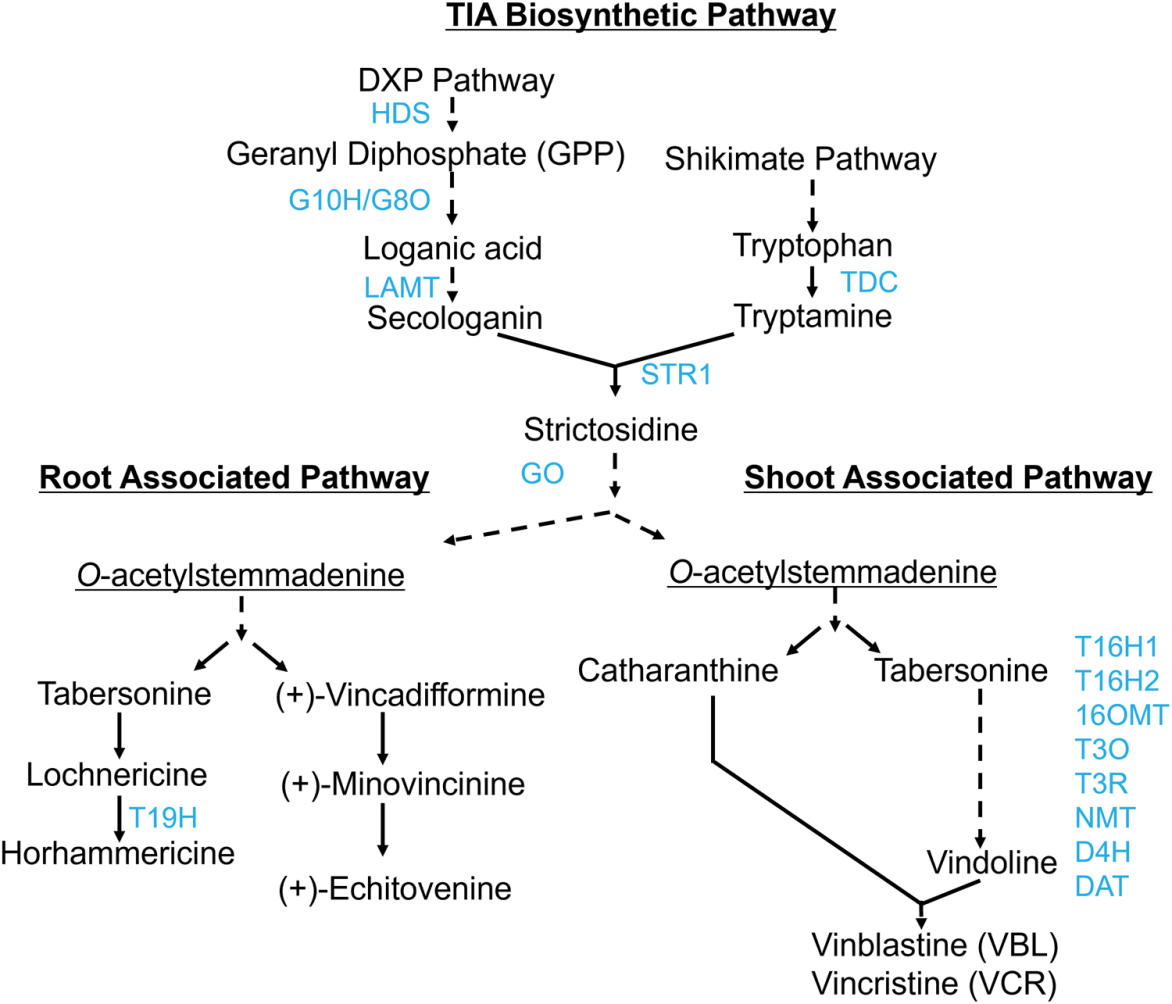
Overview of the *C. roseus* terpenoid indole alkaloid (TIA) biosynthetic pathway. The TIA biosynthetic pathway is illustrated with major metabolites named. Dashed lines indicate multistep conversions while solid lines indicate direct conversions. Select enzymes are noted along arrows in blue (color figure online)

The medicinal plant *Catharanthus roseus*, commonly known as the Madagascar periwinkle, produces the life-saving anticancer medicines vinblastine and vincristine. Vinblastine and vincristine are produced in extremely low concentrations in *C. roseus* (0.0002–0.0005% by weight), contributing to a shortage of these critical drugs (Noble 1990; Pan et al. 2016b; Rabin 2019; Shuman et al. 2020). Vinblastine and vincristine are produced from the terpenoid indole alkaloid (TIA) pathway, a tightly controlled pathway comprised of over 30 steps (Kulagina et al. 2022). TIA biosynthesis is initiated with the condensation of the terpenoid and indole branches to form strictosidine (Fig. 1). Strictosidine is enzymatically converted to produce over 130 different TIAs (van der Heijden et al. 2004).

While the enzymatic steps of the pathway have been elucidated, an understanding of its regulation is still emerging (Colinas et al. 2021; Liu et al. 2021). When *C. roseus* is subjected to herbivory, the jasmonate-regulated defense pathway is activated, initiating a cascade of both activating and repressing transcription factors, contributing to the tight regulation of TIA biosynthesis (Goklany et al. 2013; Peebles et al. 2009a; Rizvi et al. 2016). Here, we characterize the **Z**inc finger ***Catharanthus*** **T**ranscription factors (ZCTs), a class of putative transcriptional repressors in *C. roseus*. The expression of *ZCT1, ZCT2,* and *ZCT3* is elicited by methyl jasmonate (Chebbi et al. 2014; Goklany et al. 2013; Mortensen et al. 2019b; Pauw et al. 2004), yeast extract (Pauw et al. 2004), or auxin (Mortensen et al. 2019b). Structurally, ZCTs contain two characteristic Cys2-His2 zinc fingers and the EAR motif, one of the most common repressor domains involved in plant transcriptional regulation (Chow et al. 2023; Ciftci-Yilmaz and Mittler 2008; Liu et al. 2022; Yang et al. 2018).

Previous studies showed that ZCT1, ZCT2, and ZCT3 may play a repressive role in TIA biosynthesis. Using transient assays, the overexpression of *ZCT1, ZCT2,* and *ZCT3* in cell suspension cultures of *C. roseus* resulted in the repression of the upstream *TDC* and *STR* promoters (Pauw et al. 2004). ZCT1 and ZCT2 were also identified as repressors of the *HDS* promoter, a gene upstream of the terpenoid branch (Chebbi et al. 2014). Further evidence included negative correlations between *ZCT* expression and TIA production. For instance, elicitation of the TIA pathway with methyl jasmonate led to an increase in the expression of both activators such as *ORCA2* and *ORCA3* and repressors such as *ZCT1, ZCT2,* and *ZCT3* (Goklany et al. 2013)*;* at moderate levels of methyl jasmonate, the ratio of *ORCA* to *ZCT* levels was high and TIA production was optimized; but at high levels of methyl jasmonate, the ratio of *ZCT* to *ORCA* levels was high and TIA production was inhibited (Goklany et al. 2013)*.* Similarly, the overexpression of *ORCA3* in *C. roseus* hairy root cultures led to increased *ZCT* levels and an unexpected decrease in alkaloid production (Peebles et al. 2009a).

In this paper, we identified three additional Cys2-His2 zinc finger transcription factors (ZCT4, ZCT5, and ZCT6) in the ZCT subgroup that includes the previously identified ZCT1, ZCT2, and ZCT3*.* Since ZCT1, ZCT2, and ZCT3 are putative repressors of TIA biosynthesis, we characterized the activity of ZCT4, ZCT5, and ZCT6 to evaluate if these six ZCTs could act redundantly or compensate as repressors of the TIA pathway. We also investigated if these six ZCTs differed in their induction by characterizing their tissue-specific expression and responsiveness to methyl jasmonate. Since *ZCT*s are induced by methyl jasmonate, we investigated the role of the jasmonate-associated CrMYC2a in regulating the expression of *ZCTs*. Our characterization suggests that *ZCTs* can be induced by CrMYC2a and that they can act as regulators of TIA metabolism, potentially as an adaptative response to jasmonate elicitation.

## Materials and methods

### Identification of ZCT4, ZCT5, and ZCT6

Coding sequences of *ZCT1, ZCT2,* and *ZCT3* were aligned with blastn against the *C. roseus* transcriptome (Góngora-Castillo et al. 2012). Hits with E values < 1E-4 were considered potentially homologous *ZCT*s. This led to the discovery of three new *ZCT*s, *ZCT4, ZCT5,* and *ZCT6*. The Cra_locus_86538 was not further characterized (Figures S1) since it did not pass the threshold of an E value < 1E−4; in addition, based on an amino acid alignment, it contains only one rather than two Cys2-His2 zinc finger domains as found in the ZCTs.

### Cladogram of Cys2-His2 transcription factors in *C. roseus*

We also searched for additional Cys2-His2 zinc finger transcription factors in the *C. roseus* genome using the TFDB search tool in the Plant Transcription Factor Database (v3.0) (Jin et al. 2014). Initially, 127 Cys2-His2 zinc fingers were identified. However, correcting for isoforms yielded 65 independent protein sequences identified by the Plant TFDB; a cladogram of all 65 Cys2-His2 transcription factors encoded in the *C. roseus* transcriptome (Góngora-Castillo et al. 2012) was generated with Qiagen CLC Main Workbench 8 (Figures S1; protein alignment parameters: gap open cost = 10.0; gap extension cost = 1.0; end gap cost = free; alignment mode = very accurate, redo alignments = no; use fixpoints = no). The Neighbor Joining algorithm and Jukes-Cantor distance measure were used with the Bootstrap method (100 replicates) to generate the tree as described in “Identification of Cys2-His2 zinc finger transcription factors in *C. roseus*” section.

### Expression analysis of *ZCTs*

RNA-Seq reads were downloaded from the NCBI-SRA databases (BioProject: PRJNA252611) and processed to obtain sequencing reads in FASTQ format. Reads were processed to trim the sequencing adapter sequences and read position with low sequencing quality using Trimmomatic version 0.36 (Bolger et al. 2014). Clean reads were aligned to the *C. roseus* reference genome v2 using STAR version 2.5.2b (Dobin et al. 2013). Aligned sequences were counted using the featureCount version 1.22 package (Liao et al. 2014) in R to get the number of reads aligned to a gene feature. Read counts data were normalized to get gene expression values in FPKM (fragments per kilo base million). FPKM values for TIA pathway genes were extracted from the following CRO numbers: MYC2: CRO_T124533, ORCA2: CRO_T110365, ORCA3: CRO_T110360, BIS1: CRO_T107535, BIS2: CRO_T107539, ZCT1: CRO_T105646_ZCT1, ZCT2: CRO_T114616, ZCT3: CRO_T130011, ZCT4: CRO_T105646_ZCT4, ZCT5: CRO_T124775, ZCT6: CRO_T110996, IO: CRO_T138994, G8O: CRO_T133061, 7DLH: CRO_T106494, IS: CRO_T130026, GES: CRO_T119458, HL2: CRO_T119458, MAT: CRO_T120028, GS2: CRO_T113153, STR: CRO_T125329, GS1: CRO_T113154, 8HGO: CRO_T107879, T19H: CRO_T119486, Redox1: CRO_T129272, 7DLGT: CRO_T131714, Redox2: CRO_T132421, TDC: CRO_T125328, LAMT: CRO_T103723, GO: CRO_T127440, SLS1: CRO_T109448, HYS: CRO_T116107, SGD: CRO_T128799, T3R: CRO_T124298, T16H2: CRO_T110598, 16OMT: CRO_T110596, NMT: CRO_T111273, T3O: CRO_T113994, DAT: CRO_T120021, D4H: CRO_T127167, HL1: CRO_T139139, T16H1: CRO_T110599. Inspection of the original CRO_T105646 gene prediction from the *C. roseus* v2 genome (Franke et al. 2018) revealed that this locus was a fusion of the *ZCT1* and *ZCT4* loci. In this paper, the fused locus was manually reannotated to create the CRO_T105646_ZCT1 and CRO_T105646_ZCT4 loci used for gene expression analysis and correlation analysis. In the most recent *C. roseus* vr3 genome (Li et al. 2023), *ZCT1* and *ZCT4* were correctly annotated and matched our manually annotated CRO_T105646_ZCT1 and CRO_T105646_ZCT4 loci*.* Heatmaps were generated in R using the gplots package (Warnes et al. 2009).

### Construction of *ZCT *and *MYC2a* overexpression plasmids

Once identified, *ZCT* and *MYC2a* wild-type coding sequences were amplified from *C. roseus* complementary DNA (cDNA) in fragments, introducing synonymous A to T mutations to remove MoClo-incompatible restriction enzyme sites (Eco31I and BpiI). Fragments of coding sequences were harnessed into the universal pL-1 plasmid pAGM1311 and carried through the subsequent MoClo levels to a final level 2 destination vector pSB90. Coding sequences and associated oligonucleotides are listed in Table S1 and Table S2. Modular cloning was used to assemble the *ZCT* coding sequences under control of the strong *CaMV* 2 × 35S promoter (plasmid part # pICH51288, Engler et al. 2014) and the *Mas (A. tumefaciens)* terminator (plasmid part # pICH77901, Engler et al. 2014). The pSB90 backbone vector containing the constitutively active VirG (*virGN54D* mutation) expressed the assembled *ZCT* overexpression cassette (Mortensen et al. 2019a). The mutated *CrMYC2a* plasmid (*CrMYC2a[D126N])* was generated from the wild-type expression vector via site-directed mutagenesis (Azenta Life Sciences). Constructed vectors were confirmed by restriction enzyme digestion.

### Construction of native promoter-reporter constructs

Coding sequences for genes of interest were identified from the *C. roseus* transcriptome (Góngora-Castillo et al. 2012) and aligned by BLAST against the *C. roseus* Sunstorm Apricot genome (Kellner et al. 2015). A region of approximately 1 kilobase upstream of the start codon was amplified and cloned upstream of an intron-containing firefly luciferase coding sequence and *Ocs (A. tumefaciens)* terminator. This firefly luciferase cassette was then assembled into the multigene level 2 destination vector with an intron-containing *Renilla* luciferase gene expressed with the *Nos (A. tumefaciens)* promoter and terminator. Constructs were validated by double restriction enzyme digests. Plasmids were transformed into *Agrobacterium tumefaciens* (GV3101 (pMP90)) for use in transient evaluation. Oligonucleotides used to amplify promoter fragments from the genome and the promoter sequences may be found in Table S3 and Table S4. The construction of the native promoter-reporter plasmids for the vindoline pathway is described in Cole–Osborn et al. (Cole-Osborn et al. 2024).

### Transient overexpression assays in *C. roseus* seedlings

*Catharanthus roseus* seedlings were transiently transformed with *A. tumefaciens* containing the effector and promoter driving reporter constructs using vacuum infiltration, as previously described (Mortensen et al. 2019a, 2022). For pooled overexpression studies evaluated by either dual-luciferase or RT-qPCR, *Agrobacterium* strains containing the individual *ZCT* (*ZCT1, ZCT2, ZCT3, ZCT4, ZCT5, ZCT6*) effector plasmid, each at an OD_600_ of 0.06, were combined. For single effector trans-activation, the *Agrobacterium* strain containing the *ZCT* overexpression plasmid was infiltrated at a final OD_600_ of 0.34. In both single and pooled effector transactivation, the *Agrobacterium* strain containing the promoter driving reporter plasmid was at an OD_600_ of 0.06 to achieve an effector to reporter ratio of 6 to 1. For overexpression studies evaluated by RT-qPCR, the reporter construct was omitted. For *pZCT1* and *pZCT3* trans-activation experiments, the *Agrobacterium* strain containing the effector (CrMYC2a) and reporter expression plasmids were each infiltrated at a concentration of OD_600_ of 0.2 each for a final OD_600_ of 0.4 (1–1 ratio).

After transient overexpression through vacuum infiltration, seedlings recovered for 2 days in the dark and one day in the light prior to harvest. For dual-luciferase analysis, 10 biological replicates of 2 pooled seedlings were collected for each condition per each experiment. For RT-qPCR analysis, 5 biological replicates of 15 pairs of cotyledons were collected for each condition per each experiment.

### Relative promoter activity via dual luciferase assays

Relative promoter activity via luciferase activity was analyzed as previously described (Mortensen et al. 2019a) with the Luc-Pair™ Duo-Luciferase HT Assay Kit (Genecopeia). The plant protein extract from two pooled seedlings (20 µL) was mixed with 20 µL of each substrate solution in accordance with the kit protocol. Samples were measured in a 96-well white plate (Corning © 3693). Luminescence was measured in relative light units (RLU) with a plate reader (Bio-Tek Synergy HTX).

### RNA extraction and RT-qPCR

Tissue (leaf or seedlings) was harvested into RNAse-free 2 mL conical tubes containing 10 × 3 mm glass beads. Upon harvest, tissue was immediately flash-frozen in liquid nitrogen and stored at − 80 °C until the day of extraction. RNA was extracted using the Direct-zol RNA Miniprep Kit (SKU R2071). RNA integrity was visually confirmed through gel electrophoresis. Single stranded complementary DNA (cDNA) was then synthesized using the LunaScript® RT SuperMix Kit (NEB #E3010). Transcript levels were then monitored via RT-qPCR using SYBR Reverse Transcriptase (RT) (ABClonal). Two technical replicates were included for each sample. Reactions were run on either the Stratagene MX3000P or Bio-Rad CFX.

### Design of viral silencing fragments for *C. roseus* seedlings

The SGN VIGS design tool (vigs.solgenomics.net) (Fernandez-Pozo et al. 2015) was used to design fragments for Viral Induced Gene Silencing (VIGS) of *ZCTs* in seedlings. Coding sequences from the *C. roseus* transcriptome (Góngora-Castillo et al. 2012) were used as queries. The database “*Catharanthus roseus* v 2” was selected for reference. Parameters included: N-mer size: 23, Fragment length: 200–300 bp, mismatches 0–2. Top results were analyzed, and off-targets were entirely mitigated except in the cases of *ZCT1* and *ZCT2* which are too homologous to accommodate independent VIGS fragments. VIGS fragments were PCR-amplified from cDNA and cloned into the Golden Gate-compatible pTRV2-GG plasmid (Addgene Catalog # 105349) (Gantner et al. 2018). Oligonucleotides used to construct VIGS fragments in pTRV2 plasmids and the VIGS sequences may be found in Table S5 and Table S6.

### Viral silencing of *ZCTs* in *C. roseus* seedlings

*Catharanthus roseus* seeds (0.4 g, Vinca Little Bright Eye, NEseed Cat #19140) were surface-sterilized in 70% ethanol for 45 s followed by 10% bleach for 10 min. Seeds were triple rinsed in sterile deionized water and then submerged and agitated in a 3% vol/vol Plant Preservative Mixture (PPM, Caisson Labs) solution for 16 h overnight in the dark (25 °C, 60 RPM). Then, seeds were planted on Gamborg’s B5 medium without sucrose and germinated in the dark for approximately 7 days. After germination, seedlings were transferred to light (16 h photoperiod) for 2 days to allow for photomorphogenesis. Seedlings were then planted in soil and grown under the same 16 h photoperiod until two true leaves emerged (~ 40 days). Once developed, seedlings were infected with *Agrobacterium tumefaciens* strains harboring pTRV1 and pTRV2 viral silencing vectors.

In preparation for infecting plants, *A. tumefaciens* (GV3101 (pMP90)) cultures were grown and induced as previously described (Mortensen et al. 2019a). After induction, cultures were normalized to an OD_600_ of 4 and the strains harboring pTRV1 and pTRV2 plasmids were mixed at a 1:1 ratio. Modified tweezers were dipped into the *Agro-*containing solution and each plant was pinched two times just below the highest node. Plants were transferred to the dark for 2 days and then placed back into the 16 h photoperiod until photobleaching in the *ChLH-*silenced condition was apparent, approximately after 21 days (Figure S6). After silencing occurred, the second emerging leaf pair after the infection point was harvested, flash frozen in liquid nitrogen and stored at − 80 °C prior to RNA extraction and evaluation via RT-qPCR. Oligonucleotides used for qPCR are listed in Table S7.

## Results

### Identification of Cys2-His2 zinc finger transcription factors in *C. roseus*

We identified the Cys2-His2 zinc fingers encoded in the *C. roseus* transcriptome (Góngora-Castillo et al. 2012) using the Plant Transcription Factor Database (TFDB) (Tian et al. 2020). Our search yielded 65 transcription factors containing from one to six Cys2-His2 zinc fingers (Figure S1). The number of Cys2-His2 zinc finger-containing transcription factors in *C. roseus* is fewer than that of *Arabidopsis* (176) (Englbrecht et al. 2004), rice (189) (Agarwal et al. 2007), soybean (321) (Yuan et al. 2018), or tomato (112) (Ming et al. 2020) and is closer to that of alfalfa (58) (Pu et al. 2021).

**Fig. 2:**
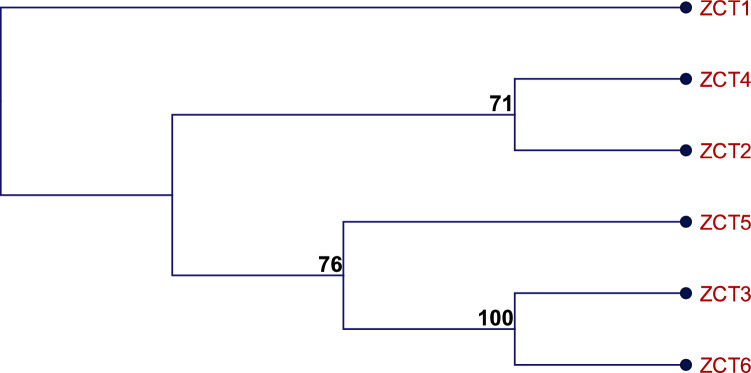
*C. roseus* ZCT gene tree based on amino acid sequence. *C. roseus* ZCTs were identified using the Plant Transcription Factor Database. The Neighbor Joining algorithm and Jukes–Cantor method were used with the Bootstrap method to generate the cladogram based on amino acid sequence alignments. Bootstrap values are indicated

Of the 65 transcription factors containing Cys2-His2 zinc fingers identified in *C. roseus*, we focused on the ZCT subgroup, which contains two Cys2-His2 zinc fingers and a specific arrangement of defining motifs (B-box, L-box, and LxLxL motifs). The ZCT subgroup consists of the previously characterized, putative repressors of the TIA pathway, ZCT1, ZCT2, and ZCT3, and the newly identified and uncharacterized ZCT4, ZCT5, and ZCT6. In the cladogram with just these six ZCTs (Fig. 2), ZCT2 and ZCT4 clustered most closely while ZCT3 and ZCT6 clustered most closely.

**Fig. 3:**
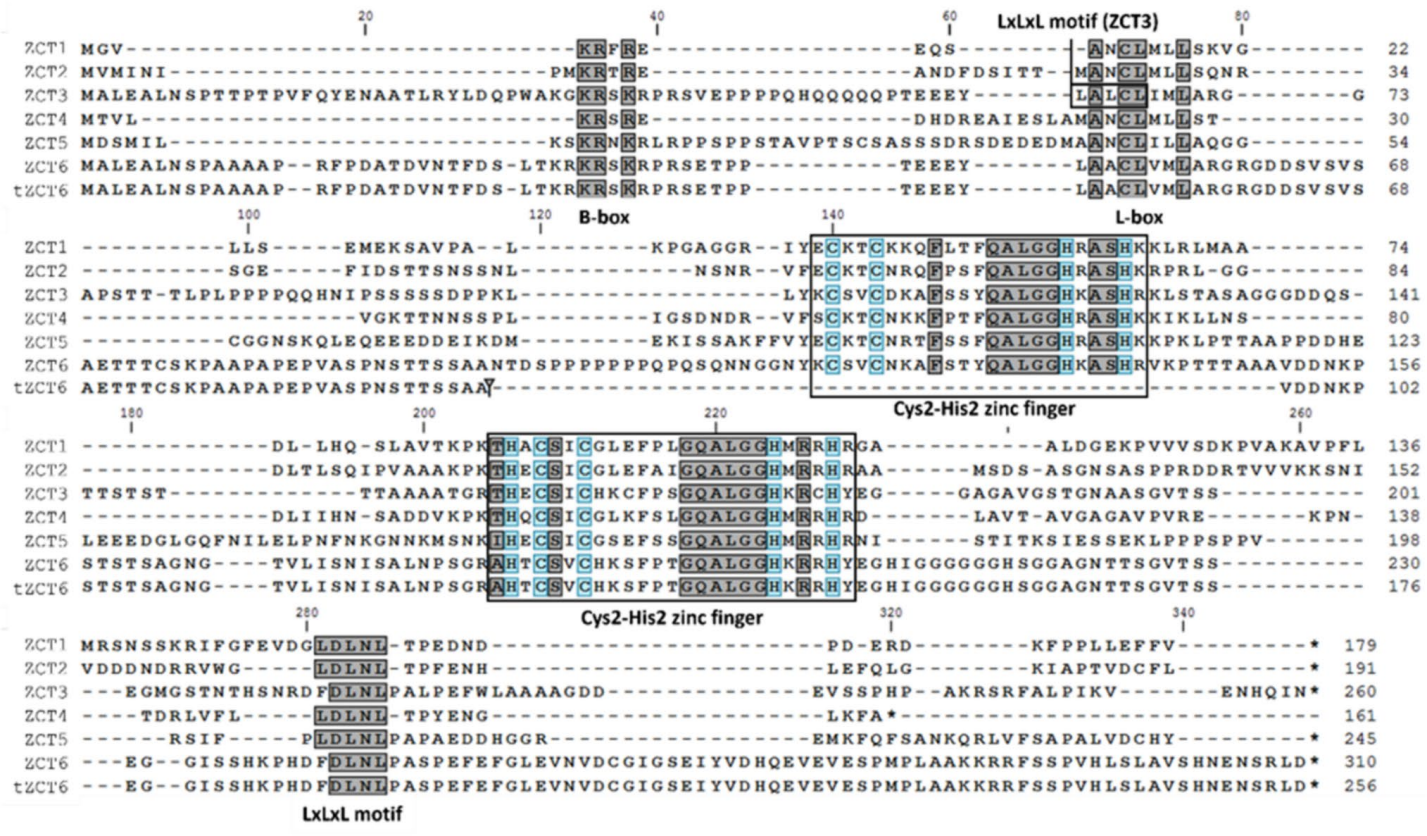
Amino acid alignment of *C. roseus* ZCT proteins shows conservation of characteristic motifs. The B-box and L-box regions are thought to play a role in nuclear localization and protein–protein interaction, respectively. The Cys2-His2 zinc fingers (with the Cys and His residues highlighted in blue) contain the QALGGH motifs responsible for binding to the major groove of the target DNA. The EAR or LxLxL motif is a strong repression domain and is consistently present in most ZCT proteins. tZCT6 refers to the truncated version of the ZCT6 protein and contains one instead of the two Cys2-His2 zinc finger domains found in the other ZCTs (color figure online)

We confirmed via an amino acid alignment that these newly identified ZCT sequences (ZCT4, ZCT5, ZCT6) contained the motifs or domains that are characteristic of the ZCT subgroup (ZCT1, ZCT2, and ZCT3) (Fig. 3). These six ZCTs share high sequence conservation mainly within their two Cys2-His2 zinc fingers but differ in the spacer length and sequence between their two Cys2-His2 zinc fingers. Cys2-His2 zinc fingers interact with target DNA through coordination with a zinc ion (Elrod-Erickson et al. 1996; Pabo et al. 2001) and through the characteristic plant specific QALGGH sequence within the Cys2-His2 zinc finger motif; the QALGGH sequence allows binding into the major groove of DNA (Takatsuji 1999). A single zinc finger provides relatively weak binding, while multiple fingers enhance binding (Iuchi 2001). In addition, the spacer length and sequence between the two Cys2-His2 zinc fingers affect the binding specificity of the ZCTs to their target (Kubo et al. 1998; Takatsuji and Matsumoto 1996). Therefore, the targets regulated by the ZCTs may differ and must be determined experimentally.

While amplifying *ZCT6* from cDNA, both a complete and a truncated transcript were identified. The complete transcript is referred to as *ZCT6* and the truncated transcript is referred to as *tZCT6* (Fig. 3). We identified two QALGGH sequences in most ZCTs, the first of which was present in ZCT1 through ZCT6 but was absent from tZCT6, likely rendering it a weaker binder; therefore, we focused on characterizing the two-fingered ZCTs*.*

Each of these ZCTs also contains a B-box which is suspected to serve as a nuclear localization signal and a leucine-rich box (L-box) which is presumed to assist in protein interaction (Kubo et al. 1998; Sakamoto et al. 2004). The final conserved element among these ZCTs is the EAR motif, a characteristic LxLxL sequence. The EAR motif is a strong repression domain and is consistently present in most of the ZCTs, with ZCT3 harboring a second LxLxL motif within its L-box. The EAR motif is one of the most common transcriptional repressor domains found in plants. This domain works by recruiting chromatin remodeling factors that prevent expression of genes (Chow et al. 2023).

A UniProt and BLAST search using the *C. roseus* ZCT protein sequences showed that the six ZCTs are most closely related to the *Arabidopsis* ZAT and AZF proteins (Table S8). The *C. roseus* ZCTs and *Arabidopsis* ZAT and AZF proteins share the highest sequence identity in the Cys2-His2 zinc finger domains but most of these *Arabidopsis* proteins did not contain all the characteristic motifs found in the *C. roseus* ZCTs (Fig. 3; Figure S2). Among the best characterized *Arabidopsis* ZATs are ZAT10 and ZAT12 (Table S8); these ZATs are involved in response to environmental stress, including hypoxia, cold, heat, salinity, drought, light, UV-B, wounding, and others. For example, ZAT10 overexpression facilitates response to osmotic stress (Mittler et al. 2006). Similarly, ZAT12 has been shown to improve tolerance to low temperature and drought stress (Davletova et al. 2005; Vogel et al. 2005). Among the Cys2-His2 zinc fingers, the *C. roseus* ZCTs (i.e., the previously characterized ZCT1, ZCT2, ZCT3) are unique in that they play a putative role in repressing alkaloid biosynthesis.

In our search for additional ZCTs, we identified ZCT4, ZCT5, and ZCT6 that clustered with the previously characterized putative repressors of the TIA pathway, ZCT1, ZCT2, and ZCT3. The ZCTs share two highly conserved Cys2-His2 zinc fingers and the EAR repression motif (with ZCT6 possessing two motifs) but differ in spacer sequence and length outside of the Cys2-His2 zinc fingers; these differences can affect the promoters they bind and regulate. Thus, we proceeded with evaluating if ZCT4, ZCT5, and ZCT6 could act redundantly or compensate as repressors of TIA biosynthesis; if the ZCTs act redundantly and are similarly induced, then increasing TIA biosynthesis would require the inhibition (i.e., knockout or silencing) of multiple ZCTs rather than one or two ZCTs (i.e., ZCT1, ZCT2), as previously demonstrated in Rizvi et al (2016).

### Tissue-specific and jasmonate-responsive expression of ZCTs

**Fig. 4:**
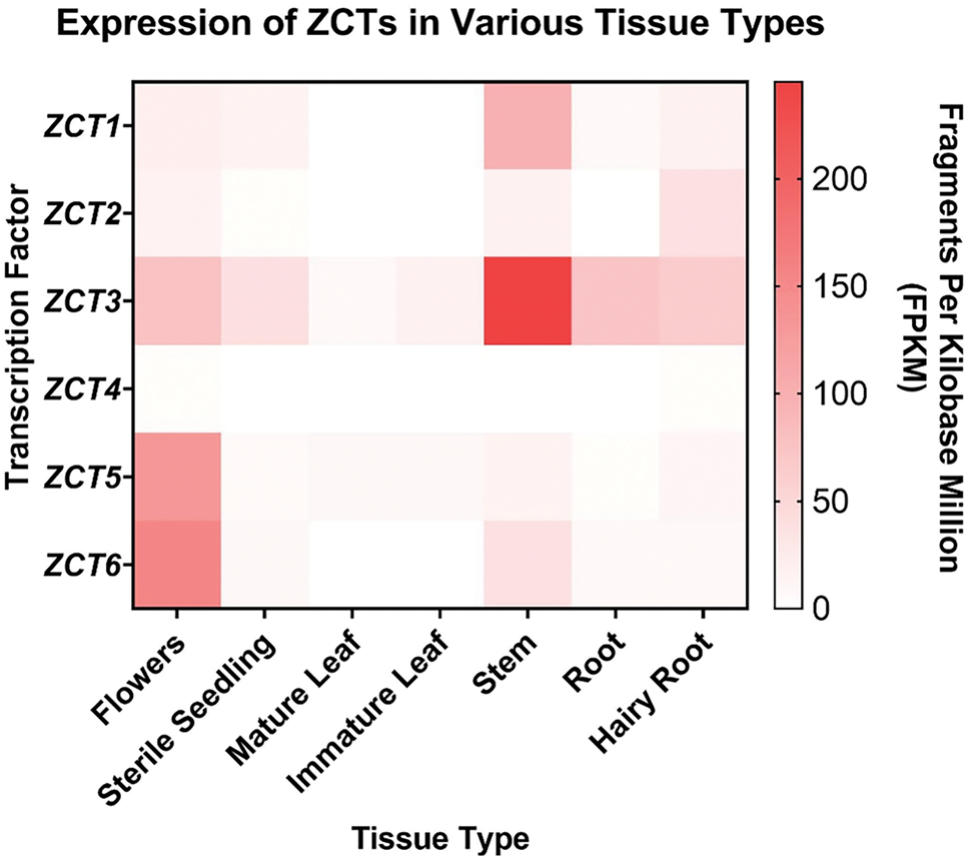
Expression of *ZCT*s in *C. roseus* tissue. Publicly available transcriptome data (Góngora-Castillo et al. 2012) was used to generate a heat map of *ZCT* expression in various tissue types. Expression level is reported as fragments per kilobase transcripts per million (FPKM)

To assess the role and redundancy of the expanded members of the *ZCT* family, we explored their expression profile in various tissue types and in response to jasmonate. We used previously published transcriptome data (Góngora-Castillo et al. 2012) to identify relative transcript levels of *ZCTs* in various tissue types (Fig. 4). *ZCT3* was the most highly expressed *ZCT* in most tissue types, except for the flower where *ZCT5* and *ZCT6* were most highly expressed*.* Flowers expressed most of the six *ZCTs* with the stem tissue being the next highest. Interestingly, the tissues that expressed the highest *ZCT* levels (flowers and stems) also contained the lowest alkaloid levels while the tissues that expressed the lowest *ZCT* levels (immature and mature leaves) contained the highest alkaloid levels (Pan et al. 2016b). This observation is consistent with the putative role of *ZCTs* as repressors of TIA biosynthesis.

Next, we evaluated the jasmonate responsiveness of *ZCT4, ZCT5,* and *ZCT6* in hairy root cultures where increasing *ZCT1, ZCT2,* and *ZCT3* levels were correlated with repressed TIA gene expression (Goklany et al. 2013; Peebles et al. 2009a). A promoter scan (Figure S3) confirmed that *ZCT4, ZCT5,* and *ZCT6* contain cis-regulatory elements that play a role in jasmonate-inducibility (i.e., WUN: wound-responsiveness; TGACG and CGTCA-motifs: methyl jasmonate responsiveness) as well as tolerance to environmental stressors (i.e., MBS: drought-inducibility; HSE: heat stress responsiveness; TC-rich repeats: defense and stress responsiveness). We subsequently determined if the newly identified *ZCT4, ZCT5,* and *ZCT6* were also jasmonate-responsive and could contribute to the repression of TIA biosynthesis associated with high jasmonate dosage in hairy root cultures, as observed with *ZCT1, ZCT2,* and *ZCT3*.

**Fig. 5:**
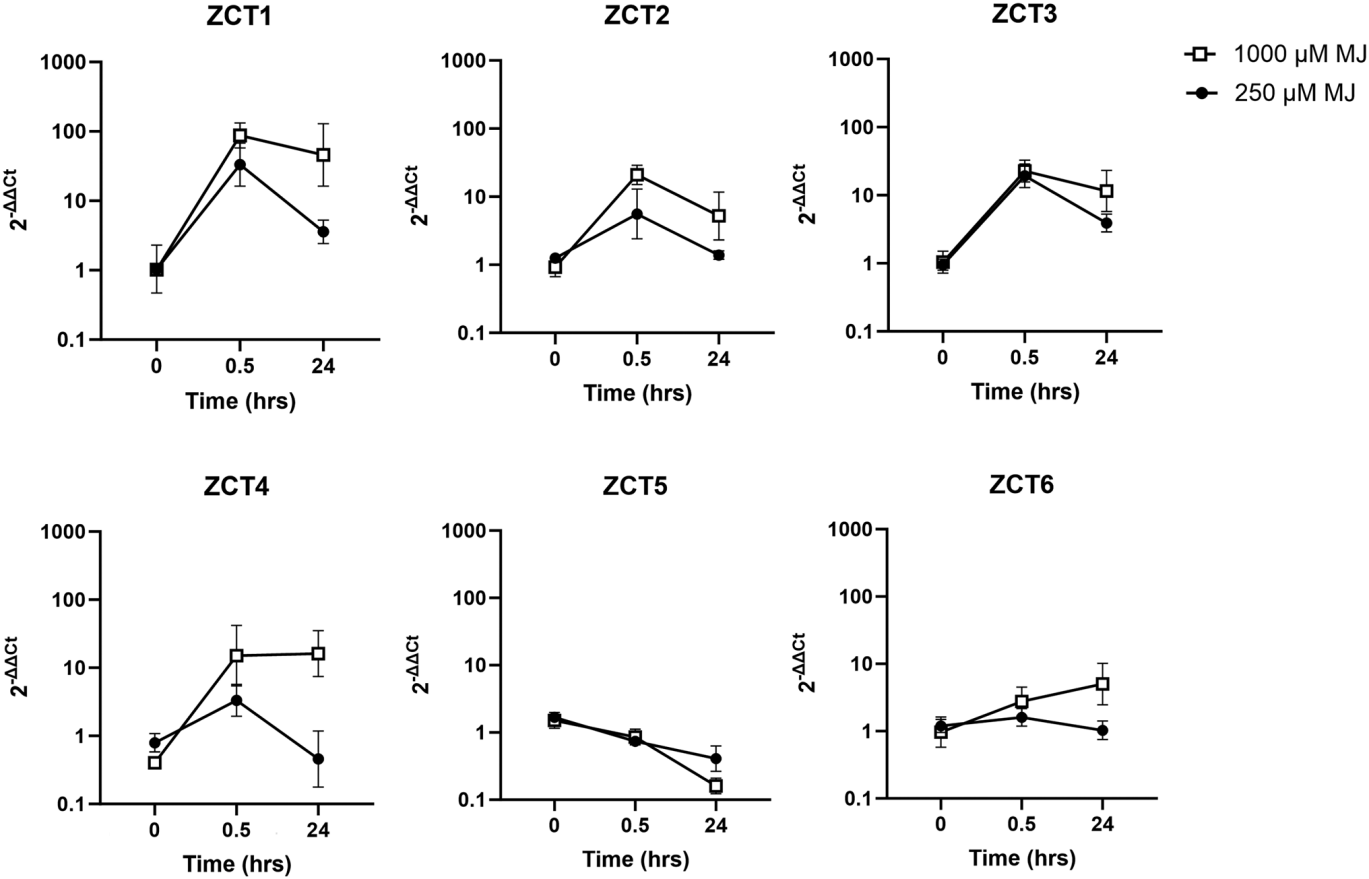
Jasmonate-responsiveness of *ZCTs* in hairy root cultures. *C. roseus* hairy root cultures were treated with two dosages of methyl jasmonate (250 or 1000 µM MJ), and tissue was collected at the designated timepoints (0, 0.5, and 24 h). Genes of interest (*ZCTs*) were monitored by RT-qPCR. Relative transcript level was first normalized to the housekeeping gene SAND and then to the control condition (0 h) using the 2^−ΔΔCt^ method. Each data point represents the average from tissue harvested from three independent flasks (i.e., three biological replicates) with error bars representing the standard deviation

We elicited *C. roseus* hairy roots with two dosages of methyl jasmonate (250 and 1000 µM MJ) and monitored *ZCT* transcript levels at 30 min and 24 h (Fig. 5). The selected jasmonate dosages of 250 and 1000 µM were previously shown to optimize and inhibit TIA pathway gene expression and TIA levels, respectively (Goklany et al. 2013). At both jasmonate dosages, all *ZCTs* (excluding *ZCT5*) exhibited a burst of expression at 30 min, a rapid response suggesting regulation by a pre-existing transcription factor. At the optimal dosage for alkaloid production in hairy roots (Goklany et al. 2013) (250 µM MJ), *ZCT1* and *ZCT3* were elevated 5–10-fold at 24 h whereas *ZCT2, ZCT4,* and *ZCT6* had returned to near basal levels. At the inhibitory dosage for alkaloid production in hairy roots (1000 µM MJ), the expression of *ZCT1* increased ~ 100-fold while the expression of *ZCT2, ZCT3, ZCT4,* and *ZCT6* increased 5–20-fold by 24 h; the high expression level of *ZCTs* was sustained at 24 h as compared to that at the optimum jasmonate dosage. Unlike other *ZCTs,* the expression of *ZCT5* was inhibited with jasmonate at both dosages. Our results are consistent with previous results that *ZCT1, ZCT2,* and *ZCT3* are induced by jasmonate. The new candidates, *ZCT4* and *ZCT6,* were also responsive to jasmonate and elevated at the high jasmonate dosage associated with repressed alkaloid production, suggesting their potential role in regulating the TIA pathway.

Finally, a co-expression analysis (Figure S4) using transcriptomic data acquired across tissues, timepoints, and treatment conditions was performed to identify TIA genes correlated with *ZCT* expression and therefore potentially regulated by ZCTs. *ZCT2, ZCT3,* and *ZCT4* were strongly correlated with each other and negatively correlated with the expression of the vindoline pathway genes. The correlation between the vindoline pathway and *ZCTs* was tested experimentally in the following section.

### The role of *ZCTs* in regulating TIA biosynthesis

To elucidate their regulatory role in the TIA pathway, the activity of ZCTs was assessed through three complementary approaches: transient co-expression of *ZCTs* with promoter-driving reporter constructs, transient overexpression of *ZCTs* with transcript monitoring, and transient silencing of *ZCTs* through viral induced gene silencing (VIGS) followed by transcript monitoring. To evaluate gene function, we used several transient expression methods since the development of transgenic *C. roseus* plants is time-intensive and has low transformation efficiencies (Choi et al. 2004; Kumar et al. 2018; Pan et al. 2012; Sharma et al. 2018; Verma and Mathur 2011; Wang et al. 2012).

**Fig. 6:**
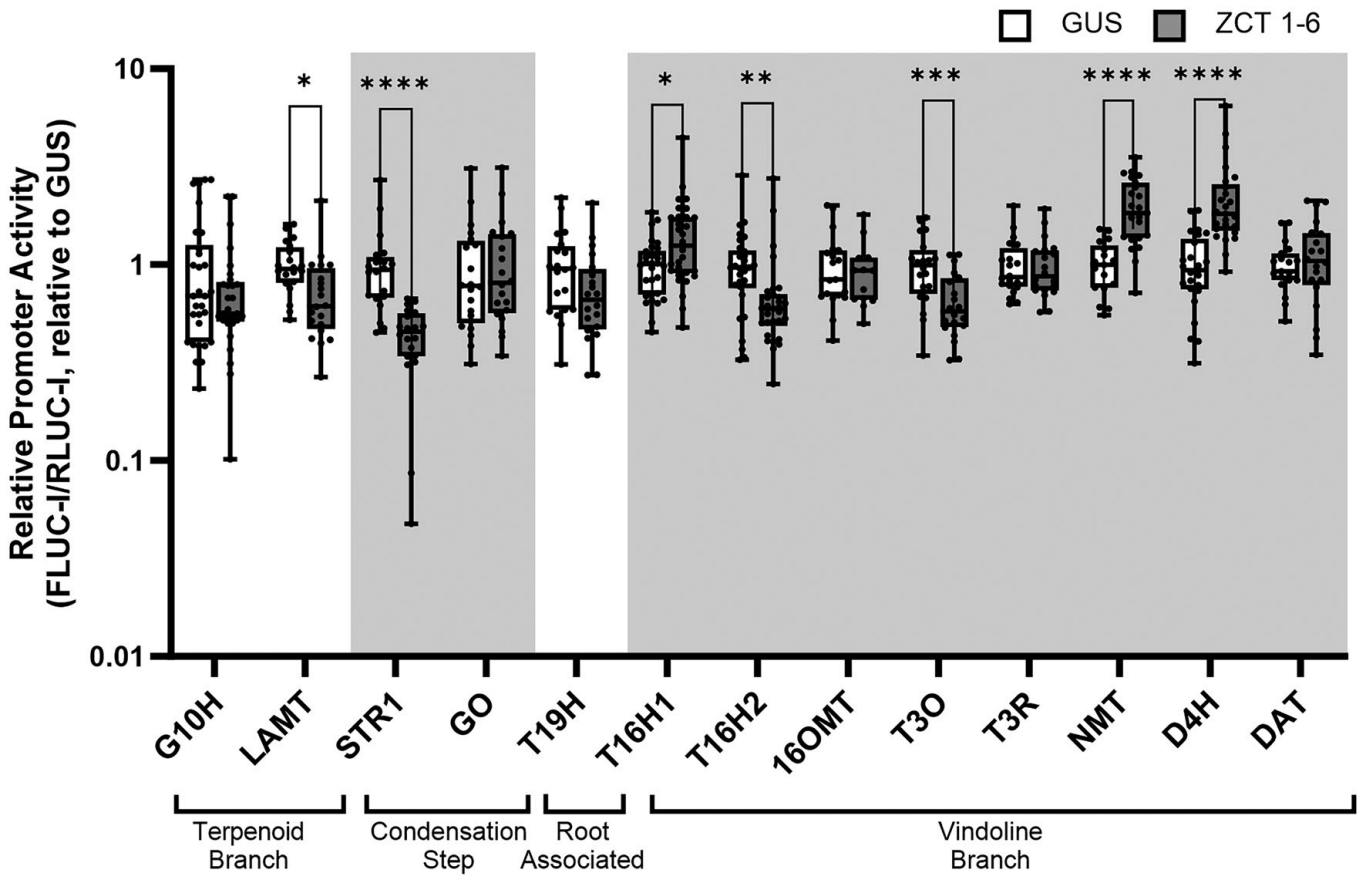
Promoter trans-activation screen of TIA pathway promoters by pooled ZCTs. Candidate transcription factors (*ZCT1* through *ZCT6*) harbored in individual cultures of *Agrobacterium tumefaciens* were pooled and overexpressed in the presence of the promoter-driving reporter plasmid with a 6 to 1 ratio of effector to promoter::reporter for a total OD_600_ of 0.4. Each biological replicate or data point consists of two seedlings that were pooled and flash-frozen in liquid nitrogen. Proteins were extracted and quantified by measuring the luminescence of the intron-containing firefly luciferase (FLUC-I) and *Renilla* luciferase (RLUC-I). Relative promoter activity was calculated by normalizing (FLUC-I/RLUC-I)_ZCTs_/(FLUC-I/RLUC-I)_Average GUS_, where GUS served as the control condition and was set to 1. The graph combines one to three replicate experiments with 7–15 biological replicates for each promoter per experiment. The horizontal line in the box plot represents the median, the ends of the box represent the 1st and 3rd quantile, and the error bars represent the range. The data were log-transformed to achieve a normal distribution prior to statistical testing. Significance based on Student’s *t* test is denoted as (*) *p* < 0.05, (**) *p* < 0.01, (***) *p* < 0.001, and (****) *p* < 0.0001

*ZCTs* were transiently co-expressed with TIA promoter-driving reporter constructs using *Agrobacterium* in *C. roseus* seedlings. First, we pooled *Agrobacterium* strains overexpressing *ZCT1* through *ZCT6* and co-infiltrated *C. roseus* seedlings with *Agrobacterium* strains containing the candidate promoter-reporter constructs (Fig. 6). Promoters screened included genes from the terpenoid branch (*pG10H, pLAMT*), the condensation and upstream steps (*pSTR1, pGO*)*,* the root-associated pathway (*pT19H*)*,* and the vindoline branch (*pT16H1, pT16H2, p16OMT, pT3O, pT3R, pNMT, pD4H, pDAT*)*.* These promoters were selected based on prior evidence: repression of the terpenoid branch (Goklany et al. 2013) and the condensation step (Goklany et al. 2013; Pauw et al. 2004) and from our co-expression analysis (Figure S4) (negative correlation with the vindoline branch). A dual luciferase assay was used to quantify promoter driven reporter activity.

Significant repression of the promoter-reporter constructs of *pLAMT* (*p* < 0.05), *pSTR1* (*p* < 0.0001), *pT16H2* (*p* < 0.01), and *pT3O* (*p* < 0.001) was observed in the screens where *ZCTs* were pooled (Fig. 6). In contrast, enhanced activity of *pT16H1* (*p* < 0.05), *pNMT* (*p* < 0.0001), and *pD4H* (*p* < 0.0001) also occurred with the pooled *ZCTs* compared to the *GUS* overexpression control. To complement the promoter transactivation studies, we also overexpressed *ZCTs* in seedlings (in the absence of the promoter-driving reporter construct) and evaluated their effect on the native gene profile (Figure S5). No significant repression of the monitored genes was observed across two experimental replicates. However, a 2-fold activation of *T16H1* expression (*p* < 0.01) was apparent in one experiment where *ZCT* expression levels were particularly high (Figure S5A), consistent with the transactivation of the *pT16H1* promoter (Fig. 6).

**Fig. 7:**
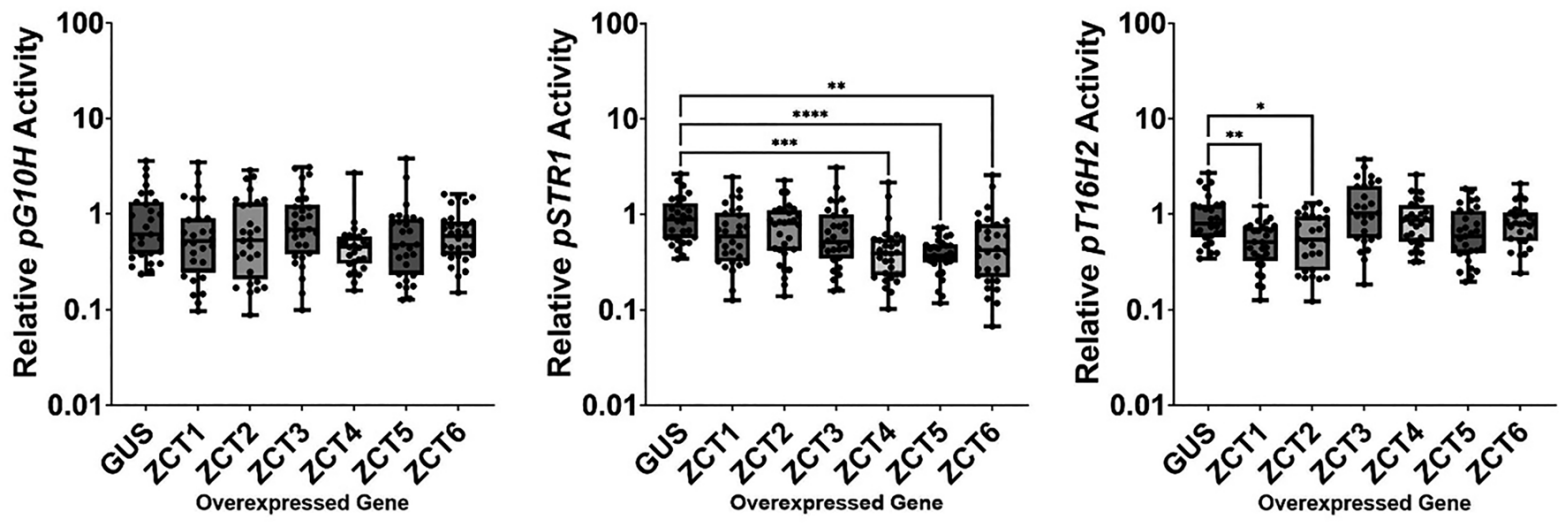
Promoter trans-activation screen of TIA pathway promoters by independently overexpressed *ZCT*s. Candidate transcription factors (either *ZCT1, ZCT2, ZCT3, ZCT4, ZCT5,* or *ZCT6*) harbored in individual cultures of *Agrobacterium tumefaciens* were independently overexpressed in the presence of the promoter-driving reporter plasmid with a 6 to 1 ratio for a total OD_600_ of 0.4. Each biological replicate or data point consists of two seedlings that were pooled and flash-frozen in liquid nitrogen. Proteins were extracted and quantified by measuring the luminescence of the intron-containing firefly luciferase (FLUC-I) and *Renilla* luciferase (RLUC-I). Relative promoter activity was calculated by normalizing (FLUC-I/RLUC-I)_*ZCTs*_/(FLUC-I/RLUC-I)_Average *GUS*_, where *GUS* served as the control condition and was set to 1. The graph combines three replicate experiments with 10 biological replicates for each condition per experiment. The horizontal line in the box plot represents the median, the ends of the box represent the 1st and 3rd quantile, and the error bars represent the range. Data were log-transformed to achieve a normal distribution prior to statistical testing. Significance based on one-way ANOVA and Dunnett’s test is denoted as (*) *p* < 0.05, (**) *p* < 0.01, (***) *p* < 0.001, and (****) *p* < 0.0001

Similarly, we performed trans-activation experiments with individually expressed *ZCT*s on a subgroup of candidate promoters across the TIA pathway (Fig. 7). We observed significant repression of *pSTR1* by *ZCT4* (*p* < 0.001), *ZCT5* (*p* < 0.0001), and *ZCT6* (*p* < 0.01). We also identified *ZCT1* (*p* < 0.01) and *ZCT2* (*p* < 0.05) to be significant repressors of *pT16H2.* Consistent with the pooled evaluation strategy, no effect on *pG10H* expression was observed with the overexpression of individual *ZCTs*.

To evaluate their role *in planta*, we performed Viral Induced Gene Silencing (VIGS) of the *ZCT*s in young plants (Figure S6). Because ZCTs repressed specific TIA promoters in our trans-activation experiments, the VIGS of *ZCTs* would be expected to upregulate these target genes. To account for possible compensation by redundant *ZCTs*, we designed 200 bp VIGS fragments that would either target individual *ZCTs* per experiment (i.e., *ZCT1/2* or *ZCT3*), clusters of *ZCTs* per experiment (jasmonate responsive cluster of *ZCT1, ZCT2,* and *ZCT3*), or all six *ZCTs* simultaneously per experiment (*ZCT1* through *ZCT6*)*.* Transcript levels were monitored via RT-qPCR and compared to that of a control condition that targeted *GFP*. Effective silencing was confirmed using a *ChLH* silencing fragment that resulted in photobleaching.

Although there was successful photobleaching of *ChLH* plants, no silencing of *ZCT*s was evident. The resiliency of the *ZCTs* to RNA silencing machinery in transiently transformed plants suggests a potential feedback mechanism. For instance, we previously observed the repression of the *pZCT1* promoter with the overexpression of *ZCT1* (Mortensen et al. 2019b). A similar resistance to viral silencing of a transcription factor was observed with the *C. roseus BIS1* VIGS experiments (Van Moerkercke et al. 2015). In a later publication, BIS was found to amplify its own expression (Schweizer et al. 2018), which would explain the difficulty of silencing BIS.

### CrMYC2a can induce the jasmonate-responsiveness of *ZCTs*

**Fig. 8:**
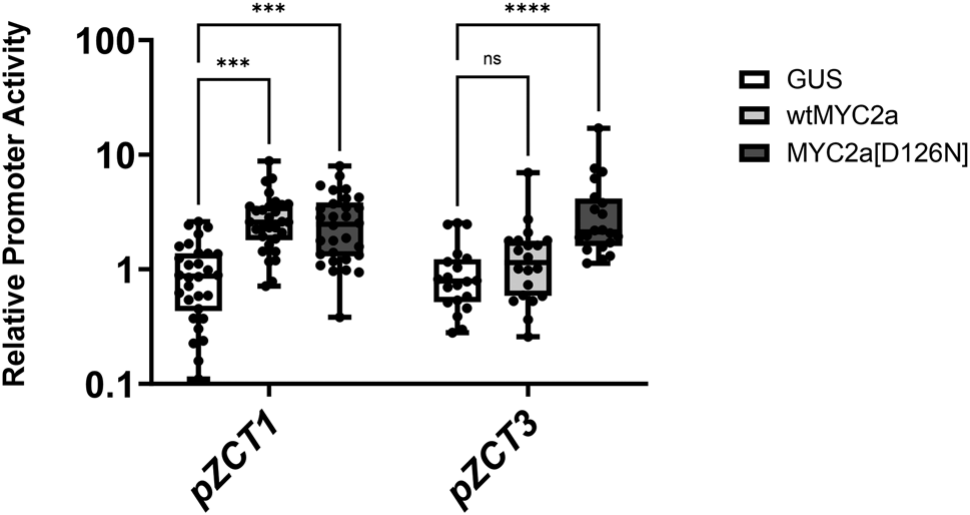
Trans-activation of *ZCT1* and *ZCT3* promoters with *CrMYC2a. GUS* (control), wild-type *CrMYC2a* (*wtMYC2a*), and mutated *CrMYC2a* (*MYC2a[D126N]*), harbored in individual cultures of *Agrobacterium tumefaciens,* were overexpressed in the presence of the *ZCT1* and *ZCT3* promoter-driving reporter plasmids with a 1 to 1 ratio for a total OD_600_ of 0.4. Each biological replicate or data point consists of two seedlings that were pooled and flash-frozen in liquid nitrogen. Proteins were extracted and quantified by measuring the luminescence of the intron-containing firefly luciferase (FLUC-I) and *Renilla* luciferase (RLUC-I). Relative promoter activity was calculated by normalizing (FLUC-I/RLUC-I)_MYC2a_/(FLUC-I/RLUC-I)_Average *GUS*_, where *GUS* served as the control condition and was set to 1. The graph combines two to three replicate experiments with 10 biological replicates for each promoter per experiment. The horizontal line in the box plot represents the median, the ends of the box represent the 1st and 3rd quantile, and the error bars represent the range. Data were log-transformed to achieve a normal distribution prior to statistical testing. Significance based on one-way ANOVA and Dunnett’s test is denoted as (*) *p* < 0.05, (**) *p* < 0.01, (***) *p* < 0.001, and (****) *p* < 0.0001

Results from the promoter trans-activation experiments suggested that ZCTs act as regulators of the TIA pathway when transiently over-expressed (Fig. 6) and in addition, that *ZCTs* are highly induced with jasmonate (Fig. 5). Since *ZCTs,* like *ORCAs* (Zhang et al. 2011)*,* are rapidly induced within 30 min of jasmonate addition (Fig. 5), we hypothesized that CrMYC2a, a pre-existing jasmonate-responsive transcription factor, activates *ZCT* expression. When jasmonate is added, JAZ is ubiquitinated and degraded (Chini et al. 2007; Thines et al. 2007), releasing MYC2a to activate the expression of the *ORCAs* (Schweizer et al. 2018; Zhang et al. 2011) and *BISs* in *C. roseus* (Van Moerkercke et al. 2016). We evaluated the role of CrMYC2a in regulating *ZCTs* through a promoter trans-activation assay in transiently transformed *C. roseus* seedlings (Fig. 8).

We evaluated trans-activation of the most jasmonate-inducible *ZCT* promoters (*pZCT1* and *pZCT3)* by overexpressing *CrMYC2a* and its de-repressed mutated *CrMYC2a[D126N]* (Schweizer et al. 2018). In addition to activating the expression of the *ORCAs* and *BISs,* CrMYC2a activates the expression of *JAZ*, leading to the regeneration of JAZ and the subsequent repression of CrMYC2a, thereby limiting the activation of TIA biosynthesis (Chini et al. 2007; Thines et al. 2007). Unlike CrMYC2a, the CrMYC2a[D126N] mutant lacks the negatively charged aspartate (D), rendering it incapable of binding to JAZ; this mutation results in a constitutively active or de-repressed CrMYC2a.

Both *pZCT1* and *pZCT3* promoters were significantly activated, i.e., 5-fold increases, by overexpression of the de-repressed *CrMYC2a[D126N]*. *pZCT1* was also activated by the wild-type CrMYC2a while the *pZCT3* promoter was unaffected. Since *ZCT3* expression is higher than *ZCT1* in seedlings (Fig. 4), the *pZCT1* promoter activity may have been lower than that of the *pZCT3* promoter, and therefore its trans-activation was more sensitive and easily observed. The activation of both *pZCT1* and *pZCT3* promoters by jasmonate-associated CrMYC2a suggests a molecular mechanism by which jasmonate-responsive *ZCT* expression (i.e*., ZCT1, ZCT2, ZCT3, ZCT4, ZCT6*) can be turned on with methyl jasmonate.

## Discussion

In this study, we investigated the role of an expanded number of ZCT transcription factors as regulators of the TIA pathway in *C. roseus*. First, we performed a bioinformatic analysis and identified 65 transcription factors containing Cys2-His2 zinc fingers in the *C. roseus* genome; we focused on the characterization of the ZCT subgroup, which included the previously characterized, putative repressors ZCT1, ZCT2, and ZCT3, and the newly identified ZCT4, ZCT5, and ZCT6. These six ZCTs contained the repressive EAR motif and were responsive to jasmonate. Thus, we investigated the potential targets regulated by these six two-fingered Cys2-His2 ZCTs.

To investigate potential targets of ZCTs, we screened promoters of genes from the terpenoid branch (*pG10H, pLAMT*), the condensation and upstream steps (*pSTR1, pGO*)*,* the root-associated pathway (*pT19H*)*,* and the vindoline branch (*pT16H1, pT16H2, p16OMT, pT3O, pT3R, pNMT, pD4H, pDAT*; Fig. 1). The transient overexpression of pooled *ZCTs* in seedlings regulated the promoters of the terpenoid (*pLAMT*), condensation (*pSTR1*), and the vindoline branches of the TIA pathway (Fig. 6). The significant repression of *pLAMT* from the terpenoid branch (by 27%) was consistent with previously published results; for instance, (Chebbi et al. 2014) reported that ZCT1 and ZCT2 acted as repressors of *HDS,* involved in catalyzing the production of isopentenyl diphosphate upstream of *LAMT.*

The overexpression of pooled *ZCTs* also significantly repressed the condensation step, *pSTR1* (by 57%). By overexpressing *ZCTs* individually in seedlings, we showed that *ZCT4, ZCT5*, and *ZCT6* could repress *pSTR1* by 41–61% (Fig. 7). Pauw et al. (Pauw et al. 2004) transformed cell suspensions via particle bombardment and showed repression of *pTDC* and *pSTR1* with *ZCT1, ZCT2,* and *ZCT3* overexpression; in contrast, we observed that *ZCT1, ZCT2,* and *ZCT3* did not repress *pSTR1* in seedlings transformed via *Agro*-infiltration*.* Differences in methodology and in tissue-specific *ZCT* levels could have impacted the observed repression. For instance, particle bombardment introduces wounding to transform plant tissue, initiating jasmonate biosynthesis responses (Bidney et al. 1992) and potentially inducing *ZCT* expression. In contrast, *Agrobacterium* infection activates the salicylic acid pathways that compete with the jasmonic acid pathways (Bidney et al. 1992; León et al. 2001; Reymond et al. 2000). In addition, promoter activity or native transcript levels will differ between seedlings and cell suspensions; thus, a lack of repression may be attributed to the low activity of that promoter where further decrease in promoter activity is undetectable. The lack of repression may also be attributed to the relatively high native *ZCT* level in that tissue. For instance, native *ZCT3* levels were already high in seedlings (Fig. 4), potentially limiting the effect of overexpression as no effect was observed with *ZCT3* overexpression in our seedlings; in contrast, the native levels of *ZCT4, ZCT5,* and *ZCT6* were low in seedlings, increasing the likelihood of observing an effect with their overexpression. Therefore, while the specific ZCT responsible for repression differed between studies, the repression of *pLAMT* and *pSTR1* by the ZCT family was consistent with that in the literature.

In our study, the overexpression of *ZCTs* repressed the vindoline pathway promoters *pT3O* (by 35%) and *pT16H2* (29%) and surprisingly activated *pT16H1* (42%), *pNMT* (97%), and *pD4H* (122%) (Fig. 6). When individually tested, both ZCT1 and ZCT2 repressed *pT16H2* by 38–47% (Fig. 7). Our study is the first to investigate the role of *ZCTs* in regulating the vindoline pathway. The EAR-motif is a main feature of the ZCTs and is responsible for the recruitment of co-repressors. Therefore, the observed activation of genes such as *pT16H1, pNMT,* and *pD4H* is likely through an indirect mechanism. For example, ZCTs might repress a repressor of the vindoline genes, resulting in increased vindoline gene expression.

As a complement to the promoter trans-activation studies, we overexpressed pooled *ZCTs* in seedlings (in the absence of the promoter-driving reporter construct) and evaluated its effect on gene expression, specifically for *LAMT, STR1, T16H1, T16H2, T3O, NMT, D4H,* genes whose promoters were regulated by ZCTs in the promoter trans-activation studies. We observed varying levels of *ZCT* overexpression between experimental replicates, particularly *ZCT4* and *ZCT5* levels (Figure S5). No repression of monitored genes was observed in these two experimental replicates (Figure S5). Due to the potential stability of these monitored mRNA transcripts, repression of TIA genes may not have been detectable via RT-qPCR. In the experimental replicate where *ZCTs* were most highly overexpressed, a significant 2-fold activation of *T16H1* was observed but *NMT* and *D4H* were not (Figure S5A), potentially due to their already high basal transcript levels. As a third approach, we attempted to evaluate the role of *ZCTs in planta* through VIGS. While *ChLH-*silencing was successful, *ZCT* levels remained unaffected (Figure S6), partly due to the already low basal levels of specific *ZCTs* in immature leaves (Fig. 4).

In our study, the expression of *ZCTs* (except *ZCT5*) increased with jasmonate in a dosage-dependent manner (Fig. 5). The rapid 30-min response of *ZCTs* to methyl jasmonate was evident, like the rapid 30-min response of *ORCA3* to methyl jasmonate previously observed (Goklany et al. 2013). Therefore, we investigated a mechanistic explanation for their high induction upon jasmonate elicitation. The activator protein MYC2a is a well characterized player in jasmonate response. This constitutively expressed protein exists in a repressed state, bound by JAZ proteins. Upon elicitation by methyl jasmonate, rapid ubiquitination and degradation of JAZ proteins occurs, freeing MYC2a to activate other genes (Chini et al. 2016). Thus, the rapid response of *ZCTs* to methyl jasmonate (within 30 min) suggests regulation by MYC2a, rather than mechanisms which require synthesis from the genome level. Previously, we demonstrated that ORCA3 did not transactivate the *pZCT1* promoter (Mortensen et al. 2019b). In this paper, we confirmed significant activation of the *pZCT1* and *pZCT3* promoters by the constitutively active or de-repressed CrMYC2a, suggesting that the jasmonate-responsive expression of the *ZCTs* (*ZCT1, ZCT2, ZCT3, ZCT4, ZCT6*) is mediated by CrMYC2a (Fig. 8).

MYC2a is an activator in jasmonate signaling pathways but also serves to regulate other pathways such as salicylic acid, gibberellic acid, and auxin biosynthesis (Kazan and Manners 2013). It is possible that *ZCTs* are activated by MYC2a for environmental adaptation. This role in environmental adaptation has been observed for zinc finger proteins in several other plant species. In *Arabidopsis*, *AtZFP11* responds to jasmonate and mediates stress-responsive genes (Dinkins et al. 2003). In rice (*O. sativa*), *ZFP179* (Sun et al. 2010), *ZFP182* (Huang et al. 2007), and *ZFP245* (Huang et al. 2005, 2009) transcription factors support plant metabolism in response to salinity and drought stress. In soybean, *SCOF-1* is activated by low temperature (Kim et al. 2001). In tomato, all 112 Cys2-His2 zinc finger proteins have been identified, and are induced to various levels upon cold, heat, salinity, and osmotic stresses (Ming et al. 2020). Therefore, due to their homology with *Arabidopsis* zinc fingers, the *C. roseus ZCTs* may play a similar role in abiotic stress response.

## Conclusion

For nearly two decades, ZCT1, ZCT2, and ZCT3 have been considered putative repressors of the TIA biosynthetic pathway in *C. roseus* based on its original studies (Chebbi et al. 2014; Pauw et al. 2004). Here, we identified additional ZCTs, ZCT4, ZCT5, and ZCT6, and characterized the expanded subgroup of ZCTs (ZCT1 through ZCT6) in *C. roseus*. The structure of ZCTs (two-fingered DNA binding domains and EAR repressor domain) paired with its jasmonate-responsiveness suggests a role in regulating the defense-activated TIA pathway. In this paper, we showed that ZCTs can repress as well as indirectly activate TIA promoters when expressed at high levels and that their jasmonate-responsiveness can be mediated by CrMYC2a.

## Supplementary Information

Below is the link to the electronic supplementary material.

Supplementary file1 (DOCX 1801 KB)

## Data Availability

Datasets generated during the current study are available from the corresponding author on reasonable request.
