# Supplementary material for: Characterization of the ZCTs, a subgroup of Cys2-His2 zinc finger transcription factors regulating alkaloid biosynthesis in Catharanthus roseus
Source: Plant Cell Rep. 2024 Aug 8;43(9):209. doi: 10.1007/s00299-024-03295-8 (PMC11310244; doi:10.1007/s00299-024-03295-8)
Supplement: Supplementary file 1 — Supplementary file1 (DOCX 1801 KB) [file 299_2024_3295_MOESM1_ESM.docx]

# Supplementary Documents:

# Characterization of the ZCTs, a subgroup of Cys2-His2 zinc fingers regulating alkaloid biosynthesis in *Catharanthus roseus*

Krystyna K. F. Traverse^1^, Samuel Breselge^2^, Juliet G. Trautman^3^, Amanda Dee^3^,
Jie Wang^4^, Kevin L. Childs^4^, Carolyn W.T. Lee-Parsons^1,3,5,6^

^1^ Northeastern University, Department of Chemical Engineering, Boston, MA, USA 02115

^2^ Northeastern University, Department of Biology

^3^ Northeastern University, Department of Bioengineering

^4^ Michigan State University, Department of Plant Biology

^5^ Northeastern University, Department of Chemistry and Chemical Biology

^6^ Corresponding author. ca.lee@northeastern.edu

Krystyna K. F. Traverse, ORCID = [0009-0001-4091-4363](https://orcid.org/0009-0001-4091-4363)

Samuel Breselge, ORCID = 0000-0003-1738-7902

Kevin L. Childs, ORCID = 0000-0002-3680-062X

Carolyn W.T. Lee-Parsons, ORCID = 0000-0001-5905-1214

Supplemental Materials

A.

**
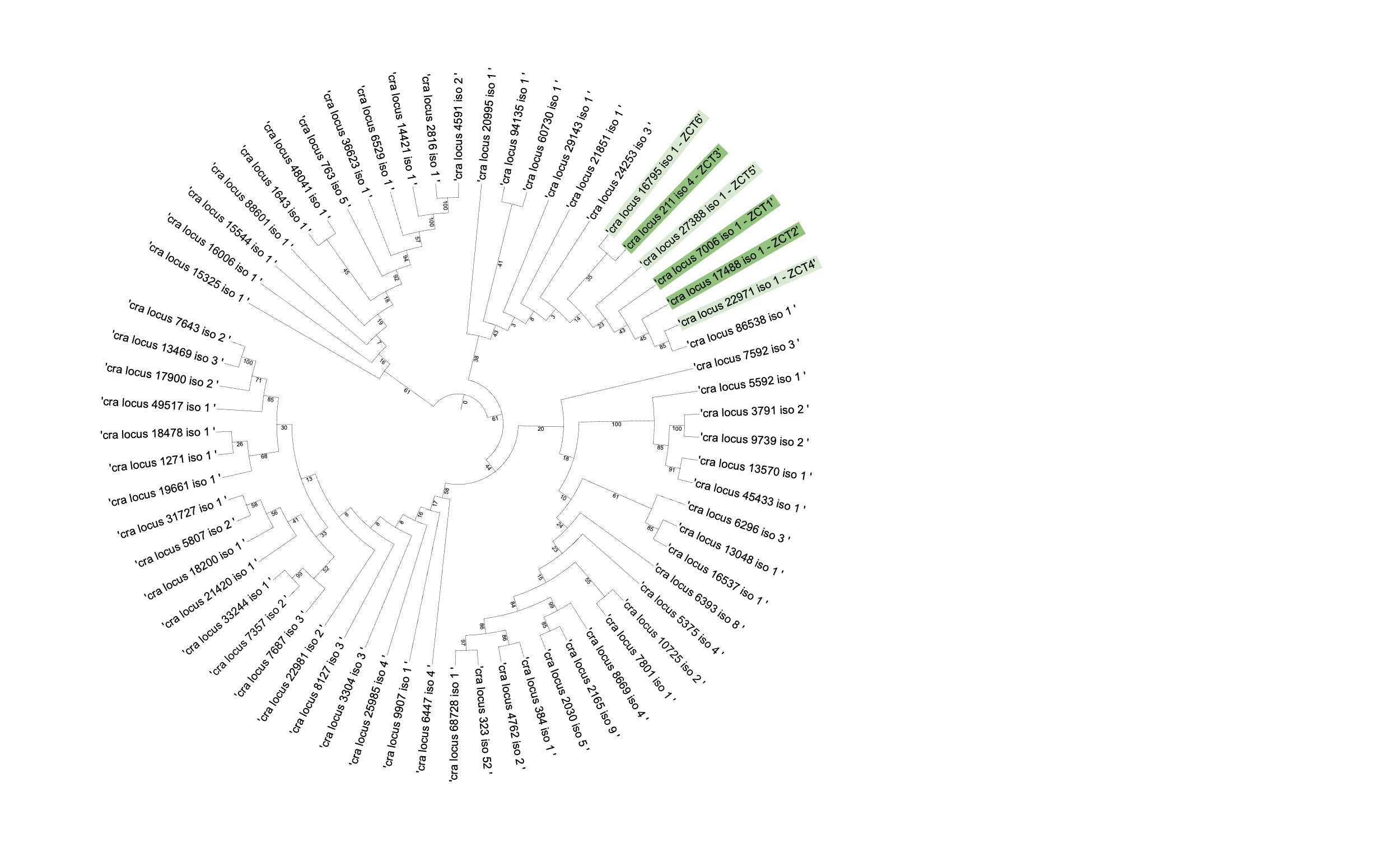
**

B.

**
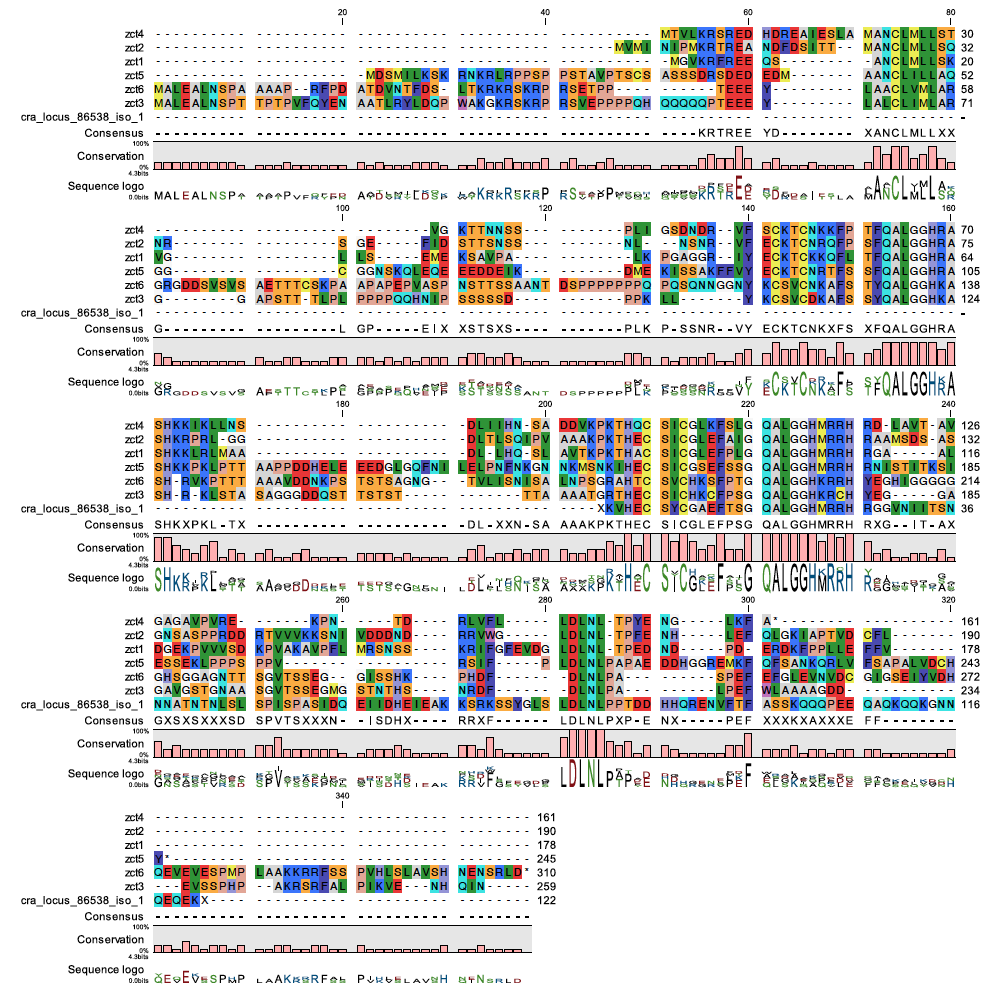
**

**Figure S1. Cladogram of C. roseus Cys2-His2 zinc finger transcription factors.** (A) The C. roseus Cys2-His2 zinc finger transcription factors were identified using the Plant Transcription Factor Database. ZCT1, ZCT2, ZCT3, ZCT4, ZCT5, and ZCT6 proteins are highlighted in green. The tree is displayed as circular cladogram with bootstrapping values. (B) An amino acid alignment of the subclade containing ZCTs and cra_locus_86538 shows that cra_locus_86538 contains only one rather than two Cys2-His2 zinc fingers as found in the other ZCTs.

***
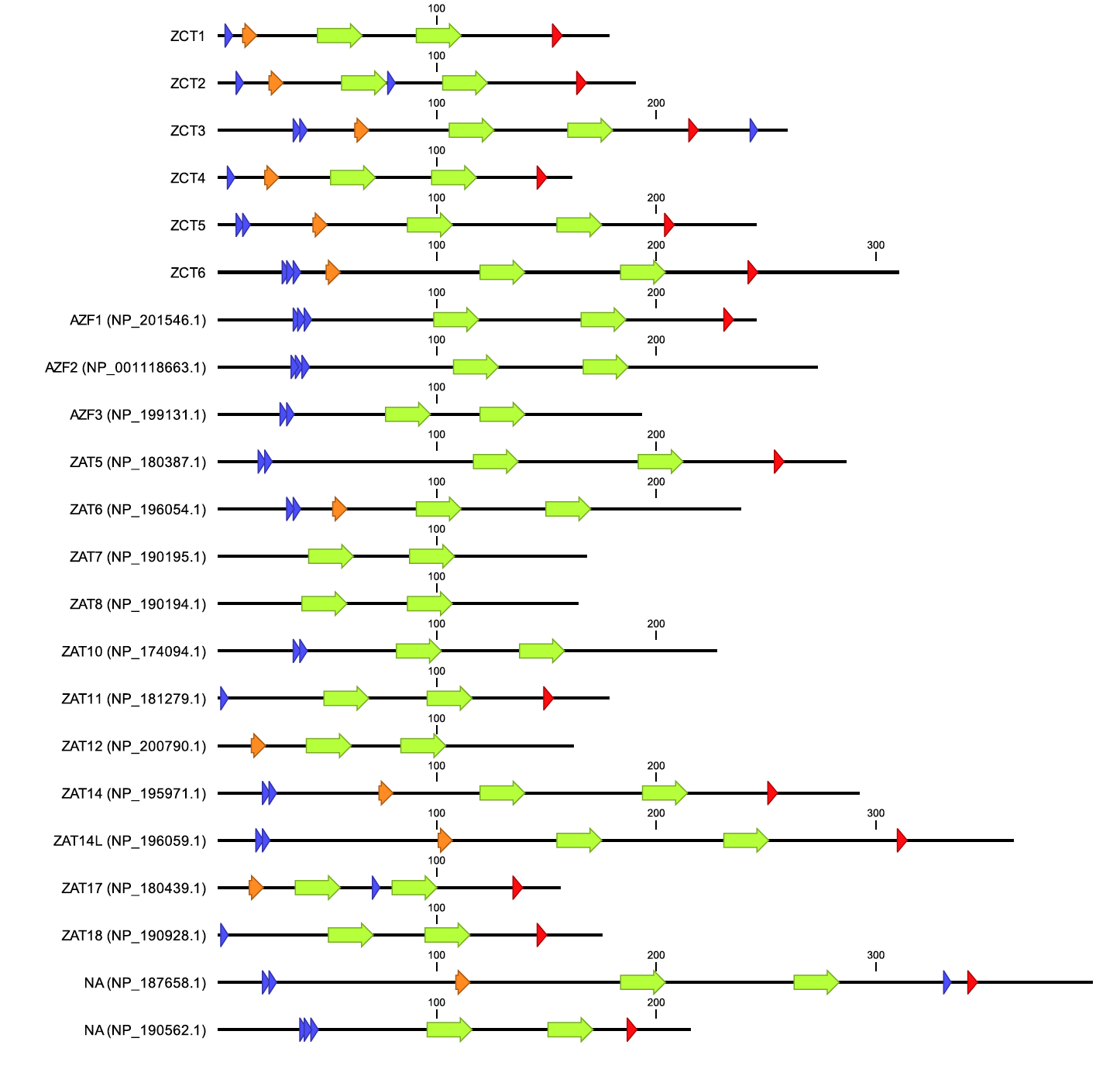
***

**Figure S2. C. roseus ZCTs transcription factors and top Arabidopsis blast hits.** Illustrated are the protein domains or motifs characteristic in C. roseus ZCT protein sequences and their top hits in Arabidopsis from UniProt and blast search (see Table S8). The highlighted motifs include the B-box (blue; Prosite motif sequence: K-R-.-[RK]), L-box (orange; Prosite motif sequence: A-[NLA]-C-L-[MIV]-[LM]-L), Cys2-His2 zinc finger (lime green; Prosite motif number PS00028), and EAR or LxLxL motif (red; Prosite motif sequence: [LF]-D-L-N-L) (see Figure 3).


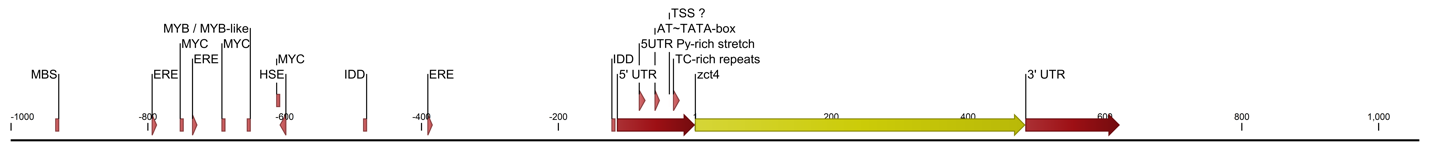


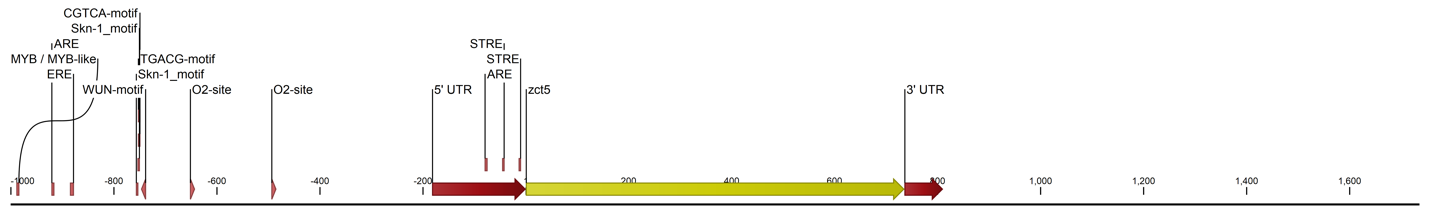


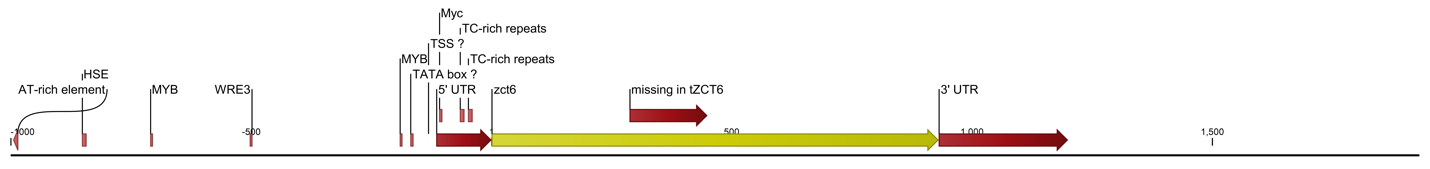


**Figure S3. A promoter scan of ZCT4, ZCT5, and ZCT6 shows stress and defense-related elements.**

Newly identified gene sequences for ZCT4, ZCT5, and ZCT6 are annotated with 5’ UTR and 3’UTR locations. Yellow sequences comprise the coding sequences. Annotations for possible motifs of stress and defense-related elements are as follows: ZCT4: MBS: MYB binding site involved in drought-inducibility; ERE: ethylene-responsive element; HSE: cis-acting element involved in heat stress responsiveness; HSE: cis-acting element involved in heat stress responsiveness; 5UTR Py-rich stretch: cis-acting element conferring high transcription levels; TC-rich repeats: cis-acting element involved in defense and stress responsiveness. ZCT5: WUN-motif: wound-responsive element; ERE; ARE: cis-acting regulatory element essential for the anaerobic induction; Skn-1_motif: Skn-1_motif: cis-acting regulatory element required for endosperm expression; TGACG-motif and CGTCA-motif: cis-acting regulatory element involved in the methyl JA-responsiveness; O2-site: cis-acting regulatory element involved in zein metabolism regulation. ZCT6: AT-rich element: binding site of AT-rich DNA binding protein (ATBP-1); HSE; TC-rich repeats: cis-acting element involved in defense and stress responsiveness. The promoter scan of ZCT1, ZCT2, and ZCT3 are provided in the Supplements of Mortensen et al (2019) (Mortensen, Weaver, et al., 2019).


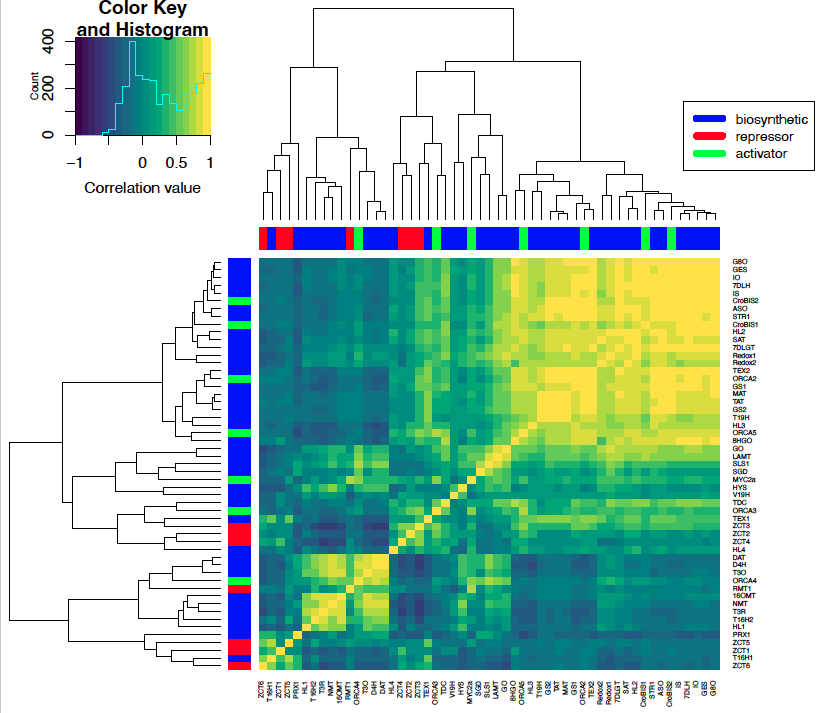


**Figure S4. Co-expression analysis of ZCTs with C. roseus genes.** TIA genes and transcription factors were evaluated for co-expression with ZCTs. RNA-Seq reads were downloaded from the NCBI-SRA databases (BioProject: PRJNA252611) and processed, trimmed, and aligned to the C. roseus reference genome v2. Read counts data were normalized to obtain gene expression values in FPKM (fragments per kilo base million). Heatmaps were generated in R using gplots package (Warnes et al., 2009).


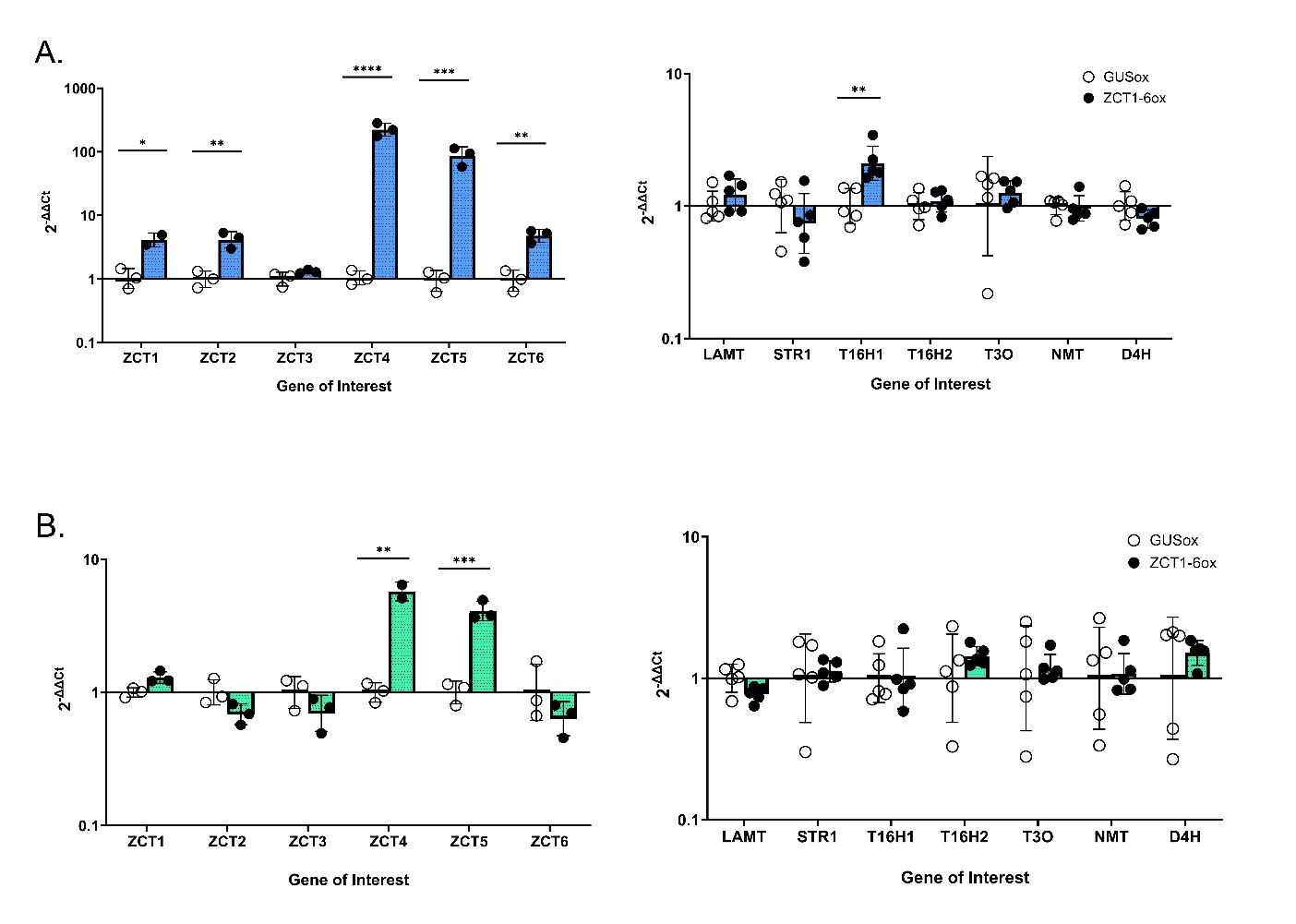


**Figure S5. Screening the expression of TIA pathway genes with the overexpression of pooled ZCTs**. Candidate ZCT transcription factors harbored in individual cultures of Agrobacterium tumefaciens were pooled and overexpressed in C. roseus seedlings. Each data point or biological replicate represents fifteen pairs of cotyledons that were pooled and flash-frozen in liquid nitrogen. RNA was extracted and genes of interest were monitored by RT-qPCR. (A.) Experimental Replicate 1 and (B.) Experimental Replicate 2 are shown. A subset of biological replicates (3) was first monitored to check for elevated ZCT levels. All 5 biological replicates were analyzed for changes in TIA gene expression (LAMT, STR1, T16H1/2, T3O, NMT, and D4H). Relative transcript level was first normalized to the housekeeping gene (SAND) and then to the control condition (GUS overexpression) using the 2^-ΔΔCt^ method. The bar represents the average of the 3 – 5 biological replicates and the error bars represent standard deviation. Significance based on Student’s t-test is denoted as (*) p<0.05, (**) p<0.01, (***) p<0.001, and (****) p<0.0001.


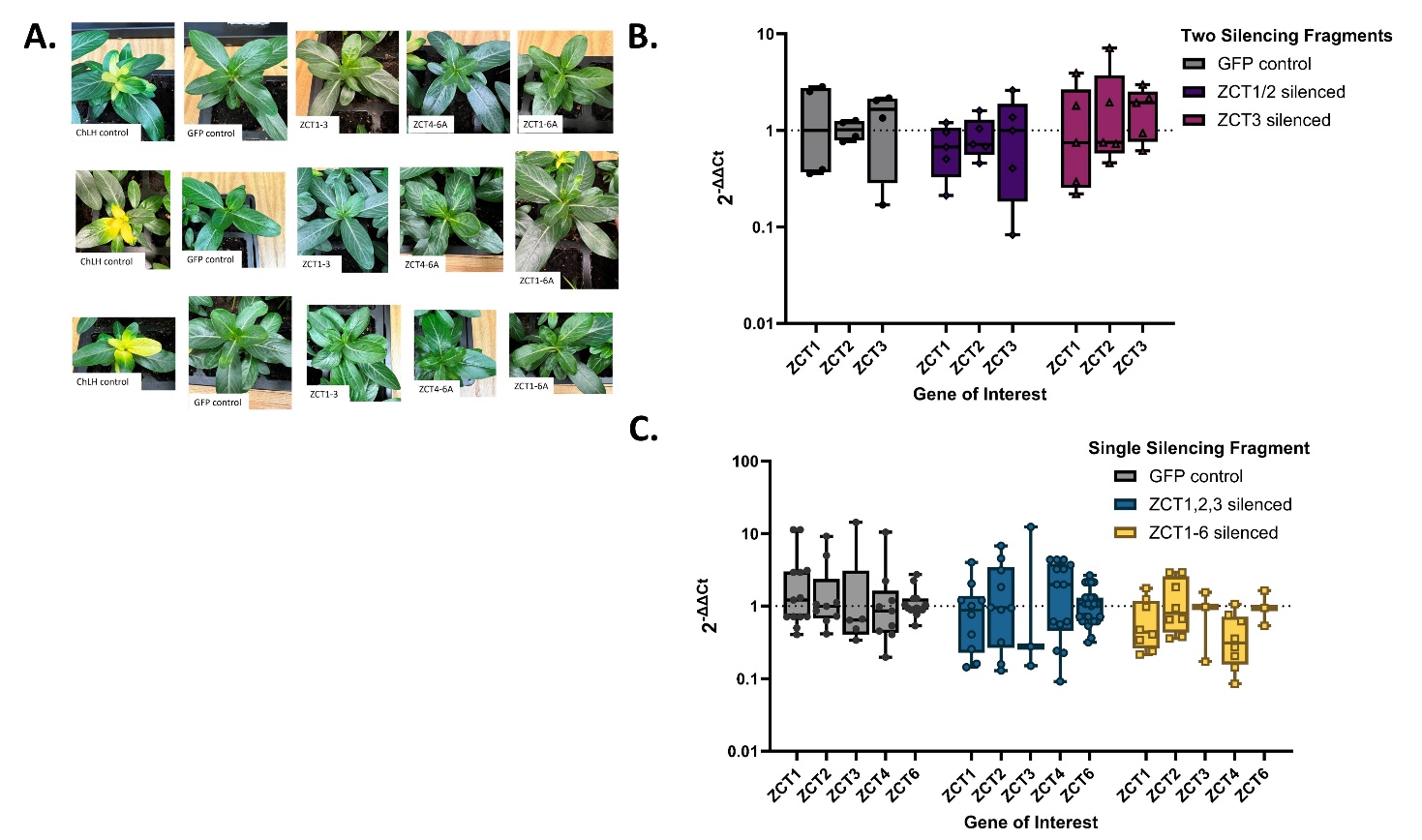


**Figure S6. Viral silencing of *ZCTs* attempted in mature leaf tissue.**

Members of the *ZCT* family were targeted by pTRV2 plasmids containing gene-specific VIGS (Viral-Induced Gene Silencing) fragments in different configurations paired with a pTRV1 plasmid. Relative transcript level was first normalized to the housekeeping gene *SAND* and then to the control condition (VIGS of *GFP*) using the 2^-ΔΔCt^ method. **A.** **Phenotypes observed through combinatorial silencing of *ZCTs* via VIGS.** Conditions pictured include: *ZCT1/2+3* silenced, *ZCT4+5+6* silenced, *ZCT1/2+3+4+5+6* silenced, and control condition, *GFP* silenced. Conditions where *ZCT4+5+6* were silenced were not pursued for RT-qPCR analysis after non-significant *ZCT* silencing was observed in other conditions. No apparent differences in phenotype were observed in comparison to the GFP control condition. **B. Independent silencing of *ZCTs.*** *ZCT1* and *ZCT3* were separately targeted with two VIGS fragments totaling 500 bp per gene. Due to homology, it was not possible to design a *ZCT1* VIGS fragment that would not also target the homologous *ZCT2*. Therefore, *ZCT2* was also targeted by the *ZCT1* fragment. Each box plot represents 5 biological replicates. **C. Combinatorial silencing of *ZCTs*.** Due to homology, one fragment was used to target *ZCT1* and *ZCT2* simultaneously whereas other *ZCTs* were targeted with its own fragment, totaling 5 VIGS fragments. Transcript levels of *ZCT5* were omitted from the analysis as design of qPCR oligonucleotides outside of the VIGS fragment region was not possible. Relative transcript level was normalized to the VIGS of *GFP* control. Each box plot represents 5-10 biological replicates spanning one to two replicate experiments. The horizontal line in the box plot represents the median, the ends of the box represent the 1st and 3rd quantile, and the error bars represent the range. Relative transcript level was first normalized to the housekeeping gene (*SAND*) and then to the control condition (VIGS of *GFP*) using the 2^-ΔΔCt^ method. Error bars represent standard deviation.

**Table S1. ZCT coding sequences and associated bioinformatic reference numbers.**

| CRO number  (Franke et al., 2018; Li, Wood, Vu, et al., 2023) | Transcript sequence ID  (Góngora-Castillo et al., 2012) | Gene | Coding Sequence (CDS) |
| --- | --- | --- | --- |
| CRO_01G008650.1  *CRO_T105646_ZCT1 | cra_locus_7006_iso_1_len_947_ver_3 | CrZCT1_CDS | ATGGGCGTGAAGAGATTCAGAGAAGAACAATCCGCAAATTGTTTGATGCTTCTTTCTAAAGTCGGATTATTATCAGAAATGGAGAAATCAGCAGTACCGGCGTTGAAACCCGGCGCCGGCGGACGAATTTATGAGTGCAAAACTTGTAAAAAGCAATTTCTCACTTTTCAGGCTTTAGGCGGTCACCGAGCGAGTCACAAGAAGCTTAGATTAATGGCGGCAGATCTACTCCATCAATCTTTAGCGGTGACGAAGCCGAAAACTCATGCTTGTTCTATATGTGGCCTCGAATTTCCATTAGGTCAGGCTCTCGGAGGTCATATGAGACGCCACCGCGGCGCCGCCTTGGATGGAGAGAAACCGGTGGTGGTGAGTGATAAACCTGTTGCAAAGGCGGTACCATTCCTGATGAGATCTAATAGCAGTAAGAGGATTTTTGGATTTGAAGTTGACGGATTGGATCTGAATTTGACGCCTGAGGACAACGATCCGGACGAGCGAGACAAGTTTCCTCCCTTACTGGAATTTTTCGTCTAA |
| CRO_08G027810.1  CRO_T114616 | cra_locus_17488_iso_1_len_958_ver_3 | CrZCT2_CDS | ATGGTGATGATTAATATACCGATGAAGCGTACGAGAGAAGCCAACGATTTCGATAGTATAACAACTATGGCGAATTGCTTGATGTTATTATCTCAAAACAGATCTGGAGAATTTATTGATTCAACAACGAGTAATTCTTCGAATTTGAATTCGAATCGGGTTTTCGAATGTAAAACATGTAATCGTCAATTTCCATCGTTTCAAGCTCTCGGCGGTCACCGAGCGAGTCATAAGCGGCCGAGATTAGGCGGAGATTTAACTCTATCGCAAATTCCGGTAGCGGCTGCGAAGCCTAAAACTCACGAATGTTCGATTTGTGGACTTGAATTCGCTATCGGTCAAGCTCTCGGAGGTCATATGAGAAGACATAGGGCGGCGATGAGTGATTCGGCGTCCGGTAATTCGGCTTCTCCTCCGCGAGATGATCGGACGGTGGTGGTGAAGAAGTCGAATATTGTTGATGATGATAATGATAGAAGAGTTTGGGGATTGGATTTGAATTTGACTCCGTTTGAAAATCATTTAGAGTTCCAGCTTGGAAAAATTGCTCCAACTGTTGATTGCTTCTTATGA |
| CRO_07G007820.1  CRO_T130011 | cra_locus_211_iso_3_len_1344_ver_3 | CrZCT3_CDS | ATGGCACTTGAAGCTTTGAATTCTCCAACAACACCTACACCTGTTTTTCAATACGAAAACGCAGCTACTCTCCGCTATCTAGATCAGCCATGGGCTAAAGGCAAACGATCCAAGAGGCCACGAAGCGTCGAACCACCTCCTCCCCAACACCAACAGCAACAACAACCCACCGAAGAAGAATACCTCGCTCTCTGTCTTATTATGCTTGCTCGCGGCGGAGCTCCATCAACTACTACTCTTCCTCTTCCACCTCCGCCGCAGCAACACAATATTCCTTCTTCTTCTTCTTCAGATCCTCCTAAGCTTCTCTACAAGTGTTCCGTTTGTGATAAGGCCTTTTCTTCTTACCAAGCACTCGGAGGACATAAGGCAAGTCACAGGAAACTCTCCACAGCCTCCGCCGGAGGTGGAGATGACCAGTCAACGACTTCAACTTCCACAACTACCGCTGCCGCCGCTACCGGAAGGACTCACGAGTGTTCCATTTGTCATAAGTGTTTTCCTTCTGGACAAGCTTTGGGAGGACACAAGCGTTGCCACTACGAAGGCGGCGCCGGCGCCGTCGGAAGTACCGGAAACGCGGCTAGCGGAGTTACTTCATCGGAAGGGATGGGCTCTACTAATACACACAGCAACCGGGACTTCGATTTGAACTTACCTGCCTTGCCGGAATTTTGGCTTGCCGCTGCAGCCGGAGACGATGAGGTTTCGAGTCCTCATCCAGCGAAACGATCCCGTTTTGCTCTTCCGATCAAAGTTGAAAACCATCAAATTAATTAG |
| CRO_01G008640.1  *CRO_T105646_ZCT4 | cra_locus_22971_iso_1_len_370_ver_3 | CrZCT4_CDS | ATGACAGTACTGAAAAGAAGCAGAGAAGATCACGATAGGGAGGCCATTGAATCCTTAGCCATGGCAAATTGTTTGATGCTTTTATCAACTGTGGGCAAAACTACTAATAATTCTTCTCCTTTAATAGGATCAGATAATGATCGAGTTTTCTCGTGCAAAACATGTAACAAAAAGTTTCCGACATTTCAAGCATTAGGAGGACACAGAGCAAGTCACAAAAAGATTAAACTTCTGAATTCCGACCTTATAATTCACAATTCTGCTGATGATGTTAAGCCTAAGACACACCAGTGTTCCATATGTGGCCTTAAATTCTCTTTAGGACAAGCTCTTGGCGGACATATGAGGCGACACCGTGACTTGGCCGTCACGGCAGTAGGAGCAGGAGCAGTGCCAGTTAGGGAAAAACCAAATACAGATAGACTTGTGTTCTTACTAGATTTAAATTTAACTCCTTATGAGAATGGTTTGAAATTTGCCTAG |
| CRO_01G011290.1  CRO_T124775 | cra_locus_27388_iso_1_len_1003_ver_3 | CrZCT5_ CDS | ATGGATTCCATGATCCTAAAATCCAAGCGAAACAAGCGCCTTAGGCCTCCTTCTCCGCCTTCTACAGCCGTTCCAACGTCTTGCTCTGCCTCTTCCAGTGACCGCAGTGATGAAGATGAAGACATGGCCGCCAATTGCTTAATTCTATTAGCACAAGGCGGCTGCGGCGGCAATTCAAAGCAATTAGAACAAGAAGAAGAAGATGATGAAATCAAGGATATGGAAAAAATTAGTAGCGCGAAATTCTTCGTTTACGAGTGTAAAACTTGTAATCGAACTTTTTCCTCTTTTCAAGCATTGGGTGGGCACAGGGCAAGTCATAAGAAGCCTAAGCTGCCGACCACAGCAGCCCCTCCCGATGATCACGAATTAGAGGAAGAAGATGGATTAGGGCAATTTAATATATTGGAGTTGCCAAATTTTAACAAAGGAAATAATAAGATGAGTAATAAGATTCATGAGTGTTCAATTTGTGGTTCTGAATTTTCATCAGGACAAGCTTTGGGTGGTCATATGAGAAGACATAGAAATATTTCTACAATTACAAAATCTATTGAATCATCGGAGAAGCTGCCGCCACCGTCACCGCCGGTTAGAAGTATTTTTCCATTGGATCTTAATTTGCCGGCACCGGCGGAAGATGATCATGGCGGCAGGGAAATGAAGTTTCAGTTTTCAGCCAATAAACAGCGTCTTGTATTCTCAGCCCCTGCTTTGGTGGATTGCCATTACTAA |
| CRO_03G026320.1  CRO_T110996 | cra_locus_16795_iso_1_len_1314_ver_3 | CrZCT6_ CDS | ATGGCTCTTGAAGCCTTGAATTCTCCAGCGGCAGCAGCTCCTCGATTTCCAGACGCTACCGACGTTAATACTTTCGATTCTTTGACGAAAAGAAAGCGTTCAAAACGTCCTCGTTCTGAAACTCCGCCGACTGAAGAAGAATATTTGGCTGCCTGTCTCGTCATGCTCGCTCGCGGTCGCGGCGATGATTCTGTATCGGTTTCCGCCGAAACCACTACTTGTTCCAAACCAGCGGCACCTGCGCCTGAGCCTGTCGCTTCTCCGAATTCAACTACCTCCTCCGCCGCGAATACAGACTCACCACCGCCTCCGCCTCCGCCGCAACCGCAATCGCAGAACAACGGTGGTAATTATAAGTGTAGCGTATGCAATAAGGCTTTCTCTACTTATCAAGCTTTAGGCGGACACAAAGCAAGCCACCGCGTAAAACCTACTACTACCGCCGCCGCTGTCGATGACAACAAGCCTTCGACATCAACCTCCGCCGGTAACGGAACGGTGCTGATTTCTAATATTTCCGCTCTCAATCCCAGCGGTAGAGCGCATACATGCTCTGTTTGTCACAAGTCTTTCCCTACCGGTCAAGCTCTCGGCGGTCACAAGCGTCGCCACTACGAAGGACACATAGGAGGCGGCGGTGGTGGTCACAGCGGAGGAGCAGGAAATACCACAAGCGGAGTGACGTCATCTGAAGGAGGTATCTCTAGTCACAAGCCTCATGATTTCGATTTGAATTTGCCTGCCTCGCCTGAATTTGAATTTGGATTGGAAGTTAACGTTGACTGCGGAATCGGAAGTGAAATTTACGTTGACCATCAGGAGGTTGAGGTTGAAAGCCCTATGCCGTTAGCGGCCAAGAAACGCCGGTTTTCTTCCCCGGTTCATCTATCATTAGCAGTATCGCACAATGAAAATTCTAGATTAGATTAG |
|  |  | CrtZCT6_CDS | ATGGCTCTTGAAGCCTTGAATTCTCCAGCGGCAGCAGCTCCTCGATTTCCAGACGCTACCGACGTTAATACTTTCGATTCTTTGACGAAAAGAAAGCGTTCAAAACGTCCTCGTTCTGAAACTCCGCCGACTGAAGAAGAATATTTGGCTGCCTGTCTCGTCATGCTCGCTCGCGGTCGCGGCGATGATTCTGTATCGGTTTCCGCCGAAACCACTACTTGTTCCAAACCAGCGGCACCTGCGCCTGAGCCTGTCGCTTCTCCGAATTCAACTACCTCCTCCGCCGCTGTCGATGACAACAAGCCTTCGACATCAACCTCCGCCGGTAACGGAACGGTGCTGATTTCTAATATTTCCGCTCTCAATCCCAGCGGTAGAGCGCATACATGCTCTGTTTGTCACAAGTCTTTCCCTACCGGTCAAGCTCTCGGCGGTCACAAGCGTCGCCACTACGAAGGACACATAGGAGGCGGCGGTGGTGGTCACAGCGGAGGAGCAGGAAATACCACAAGCGGAGTGACGTCATCTGAAGGAGGTATCTCTAGTCACAAGCCTCATGATTTCGATTTGAATTTGCCTGCCTCGCCTGAATTTGAATTTGGATTGGAAGTTAACGTTGACTGCGGAATCGGAAGTGAAATTTACGTTGACCATCAGGAGGTTGAGGTTGAAAGCCCTATGCCGTTAGCGGCCAAGAAACGCCGGTTTTCTTCCCCGGTTCATCTATCATTAGCAGTATCGCACAATGAAAATTCTAGATTAGATTAG |

* Inspection of the original CRO_T105646 gene prediction from the *C. roseus* v2 genome (Franke et al., 2018) revealed that this locus was a fusion of the *ZCT1* and *ZCT4* loci. In this paper, the fused locus was manually reannotated to create the CRO_T105646_ZCT1 and CRO_T105646_ZCT4 loci used for gene expression analysis and correlation analysis. In the most recent *C. roseus* vr3 genome (Li, Wood, Hamilton, et al., 2023), *ZCT1* and *ZCT4* were correctly annotated and matched our manually annotated CRO_T105646_ZCT1 and CRO_T105646_ZCT4 loci*.*

**Table S2.** **Oligonucleotides used to amplify and clone ZCT coding sequences**

| **Target CDS** | **Oligonucleotide Name** | **Sequence (5' to 3')** |
| --- | --- | --- |
| ZCT1 | ZCT1_mo_F | ttgaagacaaAATGGGCGTGAAGAGATTCAGAG |
|  | ZCT1_mo_R | ttgaagacaaAAGCttaGACGAAAAATTCCAGTAAGGGAGG |
| ZCT2 fragment 1 | ZCT2_mo_F1 | ttgaagacaaAATGGTGATGATTAATATACCGATGAAGC |
|  | ZCT2_mo_R1 | ttgaagacaaCTCCTCATATGACCTCCGAGAGC |
| ZCT2 fragment 2 | ZCT2_mo_F2 | ttgaagacaaGGAGACATAGGGCGGCGATG |
|  | ZCT2_mo_R2 | ttgaagacaaAAGCTCATAAGAAGCAATCAACAGTTGG |
| ZCT3 | ZCT3_mo_F | ttgaagacaaAATGGCACTTGAAGCTTTGAATTC |
|  | ZCT3_mo_R | ttgaagacaaAAGCCTAATTAATTTGATGGTTTTCAACTTTGATC |
| ZCT4 | p_zct4_f | ttgaagacttaATGACAGTACTGAAAAGAAGCAG |
|  | p_zct4_r | ttgaagacaaAAGCCTAGGCAAATTTCAAACCATTC |
| ZCT5 fragment 1 | P_zct5_p1_f | ttgaagacttaATGGATTCCATGATCCTAAAATCC |
|  | P_zct5_p1_r | ttgaagacaaATCTTCATCTTCATCACTGCGG |
| ZCT5 fragment 2 | P_zct5_p2_f | ttgaagacttAGATATGGCCGCCAATTGCTT |
|  | P_zct5_p2_r | ttgaagacaaCCTCATATGACCACCCAAAGCTTGTC |
| ZCT5 fragment 3 | P_zct5_p3_f | ttgaagacttGAGGAGACATAGAAATATTTCTACAATTACAAAATC |
|  | P_zct5_p3_r | ttgaagacaaAAGcTTAGTAATGGCAATCCACCAAAGC |
| ZCT6 | P_zct6A-f | ttgaagacttaATGGCTCTTGAAGCCTTGAATTC |
|  | P_zct6A-r | ttgaagacaaAAGCCTAATCTAATCTAGAATTTTCATTGTGCG |

**Table S3.** **Oligonucleotides used to clone C. roseus promoters.**

| **Oligonucleotide Name** | **Sequence (5' to 3')** |
| --- | --- |
| pLAMT_F_1 | aaGGTCTCaACATGGAGGCAAAAGAAAGAGAGAAAG |
| pLAMT_R_1 | ttGGTCTCtACAATGACTTCACTTCATGAATTAGTAGTACG |
| pLAMT_F_2 | aaGGTCTCaACATGTCAAAAAGATTAATAAAAATGTGAACG |
| pLAMT_R_2 | ttGGTCTCtACAATCTCGAAATCGAGCAACAG |
| pLAMT_F_3 | aaGGTCTCaACATGAGACTTCAGTTACAATGGTCAAATATG |
| pLAMT_R_3 | ttGGTCTCtACAAGCTTAATATATTTGGAAGTCTTTTGTATTAAG |
| pLAMT_F_4 | aaGGTCTCaACATAAGCCCGTAATTTATATATAACTCTTTCTTTATTAG |
| pLAMT_R_4 | ttGGTCTCtACAACATTTTTCTTTCTTCTCTGTATGAGTTTTTTG |
| pGO_F1_Frag1 | aaGGTCTCaACATGGAGCGTAATGAATTTAGTGACGGATCG |
| pGO_R1_Frag1 | ttGGTCTCtACAACAGTGGATTGCAAGCTGAGC |
| pGO_F2_Frag2 | aaGGTCTCaACATACTGTTATGAGCAGAGAAACTGTGGTAC |
| pGO_R2_Frag2 | ttGGTCTCtACAACATTGTGTTTTGTTCAAGAGAGGAG |
| pT16H2_F1_frag1 | aaGGTCTCaACATGGAGCCGGGTAGGCAGTTTGATTG |
| pT16H2_R1_frag1 | ttGGTCTCtACAACGATTTTTTCCTCACGTGGG |
| pT16H1_F1_frag1 | aaGGTCTCaACATGGAGCCGGGTAGGCAGTTTGATTG |
| pT16H1_R1_frag1 | ttGGTCTCtACAACGATTTTTTCCTCACGTGGG |
| pT16H1_F2_frag2 | aaGGTCTCaACATATCGCAAAACACAATCAAAGC |
| pT16H1_R1_frag2 | ttGGTCTCtACAACATTAGTGAAAATTATGCTTGTTATTGATTTGC |
| pT16H2_F2_frag2 | aaGGTCTCaACATATCGCAAAACACAATCAAAGC |
| pT16H2_R2_frag2 | ttGGTCTCtACAACATTAGTGAAAATTATGCTTGTTATTGATTTGC |
| T19H_F | ttgaagacaaGGAGCCATGAGGTAGAAATTATGAAAGAAATTAAGG |
| T19H_R | ttgaagacaaCATTATTTTTCTTGCTAGAGAAATCTAGTTGTTTTTGC |
| STR_pro_F | ttgaagacaaGGAGGATCTTTATTGTTTGTATTTTCATAATTAATTAATCCC |
| STR_pro_R | ttgaagacaaCATTGTTGACTGTAAATGATGGGAGTG |

**Table S4. C. roseus promoter sequences tested via transient promoter trans-activation assays**

| Promoter | Sequence (5' to 3') |
| --- | --- |
| *pLAMT* | GGAGGCAAAAGAAAGAGAGAAAGAAGAAAAAAGAAATAAGGAGGAAGAAGAAAGAGAGAAAAAAATGGGCGTGCGACTGAGGCAAAAGAAAGAGAGAAAGAAGAAAGAGAAAGAAAAAAGAGGCGGCGCGGCTAGGGTTTATAACGTAATAACGCATAGTCATTGTGTGTTATCACTCATAATAATGGACAATGGACGTGCGTTATCATGAATGATAACGCACAATGGCTGTGCGTTATTGCCACATCAGTGCCAGATCATCGATTGATAACGCACAATGGCTGTGCATTATCGTCACATCAGCGCCACATCACCGCCACGTTAAAAATGCTATTTAACGCGGCTTAACGTCTCTTAAGCTGGTGATTAGCGATAACGCACAATCATTGTGCGTTATCTGAGGTGATAACGCACAATGACCGTGCGTTATAGCCACTTTCAAATTTTAAAAAAAATATTGACCTATTTAGTACCAATTTCTTGAAATTAGGCTAAAGAAAGAAAAAACCTGTACAATACCTAAACGTGCTTCCGTCACCGGAGGTAGTAAAATTTCAGTTGTAAAAACATAAAAAAATAAAATAAAATGCATAAAAACAGAGATAATTAAAAAACAATACATATAGGATGTAGTGAATGATGGAAATTCTTTCTTTGACCAAACAAAGCTCATCATATTTGAACGCAAGAAAGTTCTCACTCGGAAGTCCAAGTGTGAAAATTCTTATATATGTTATAGGCAAATTATTTGCTGTTTTTTTTCTCCAAATACGTCACAAAAAAAAATTTAATTAATCCTTACTTATGTCTCCAAATATACTTACCTGATTTAATATTCCTTCCTTGAAAATATTAAAAATGATTTTAATTTTGACTATTTACATTAATGAATAAACATGCATTTATAGTCTATTTCGTACTACTAATTCATGAAGTGAAGTCAAAAAGATTAATAAAAATGTGAACGTTTAATTTGAAACACATGTTAGATGATATGTGTGGCCGCCGCCTGTTGCTCGATTTCGAGACTTCAGTTACAATGGTCAAATATGAAACCTGTTAATAATTCATATGAATTATTATTAAAAAAATTATTATAAAGAATTATTCAATTCATATATGTATCGATGTATGATGCGAATATATTTTTTTTTTTACCTCGCATTTTTCTGTTTTATTTGCACTTTTATCTGTTCTATTATTATTAAATTGTCACTTGTTTTATTATTAAATAATTGTCATTTGATTTATTCTTTTTAAGTAGTATTTCTCCTTTTTATTATTTTTTAAAAATAAATTAACAAATGAAAATTTGATGGTAATGGAAGTAGATATTAATATGATTATGTTTTTAATTTAAAATTTTAATTCTTTTATAAAGTAATAAATTTTATAAGATTTATAAAATTTATGTTGATATATAAAAAAAATTAATCATACACACTCATTAACTATTTTGTCCTTTTTATTCTATTAAACTATTTTTTCAAAAGTTTATAATTATAAATTATTTACAATTTTACATATATATCAAATTATACTTAATACAAAAGACTTCCAAATATATTAAGCCCGTAATTTATATATAACTCTTTCTTTATTAGGTTATAACTACACAATTAAAATTAAAATAATACATTTAAAAAATACATATCTCTATTATCGTTCTATACACATCTCCTAAAAATAATATATAGAACCCAATTATTCCTTGGTAGGTGATCATATAATAAAGGAAGGAGATTGGAAGCCGTGGAGGTTCATTCATGCAGTAAAAAAAAAAAAACCTATCTAACCATGTGCTGATTCATGTGGCTGCTAAGTAATAAAAATAAAAGAAAATTCTTAGAAGAAAAAAAAAAGAAAATTAGAACATTTATTGCCTATATAACCACTTCAGTTTGCACTCACTTCTTCATCTCTCTCTTCACAACAAAAACAGGAGAGAGATTTCTTATTCAACAAAAGCTCAAAAAACTCATACAGAGAAGAAAGAAA |
| *pT16H1* | AGTGAAAATTATGCTTGTTATTGATTTGCTTTAGGATAAAAGCCCTTACGATGCATGGCTTTTATAGGGGATTCTCTATAATTCCTTCACTTGTCTTGGTCCATATGTTGCTTTTTTGGCAATTTTTAATTTTTTTCTAACATCAATTATTTTTTGACTTATGGAGGATGTGTTTCTTCTCTAGTGGTTGGTAGTTTGGGTGGTATATGAATTTTTTAATGTGATAATTAAGTTTTAATTAAAGTAATTAAATATTTTTAGTATAAAAAATATTATCTCTAAAGCTTAAATTATTGTAAATTATATACTATATTTCATATTTTAATATTTGAGTATTTAATTTATGTTTTGGTTATGTATTTTTTAAAAAAATAAAAATTATATGAATGATTTACAAAGTTCATTTAATGCTTATGACATAATAATTATTTTTTTAAAAGATTTAGAAGTAATTTTTGATTATTTTTACCGATAATGAATGAAATGTTATTGATCAAATCATATTTAGAAACTTATATATAAGCATCATTAACACTACATCAAAAATAATTTCTTTAAATATCTGTTGAATTTTTAATAAAAAAGAAGTAAGAAAATATAAACTTTTTAATATTTAGCAAAAAATGATTAAAAAATTTATAATTTTAATATGTTTCTCATTTTTTATTTTAAAAAAATTAAATTTATTTTGATTGCCTTTCACTCTCTTGGGAAAGACAAACGAACTTTTCAACAATTAATTATCATGTTTTTGGACTCGATGAAAAAATCTTTAATGGAAATCAATGGAAAGACGCAAATTCTGCTTCTAGCCGTGCTTTGATTGTGTTTTGCGATTTTTTCCTCACGTGGGAACCGTGCTTTTATTGTCTGTTAACATATTTTTAATTATATTTCCTCCATTCAATTTATTTATGGTTGGAATATATTTTTTTCACTTTAAAATATTTTTCAATTTTAAAATACTTGTTACTTTAAAAATTAAAAGATTAATAAATGAAAATTTTTTCACACATGTTATTACTTTTTATTATTTAAATAGACGATCTTATAGAATATTTAAACATGACTCATATAAACTCATATTTTTAAATAATAACAATTTAGTCTATAAATTAATTATTTTCTTAATAATTGTATGTTAAAGTTTGTCTAACCGTATATCGAAGGGAATACATAATGTGGATTACTTTTATAATTTAATATAGTATAAATGTTCTTAAAATTCAATAATTTACATTAATTATTAAATTTTAAAAACTTTATTATACTTAAAATATTATATAGACTATGTTCTATTCATATTTAATTCATATTTAAGGCACAAGATTTTATGTACCACTTTATATATTACTCTATATGTCACATTCTTTTTATAACATATTTTTTTTAGAGTTTATTTTTTTCTTAGAATTTATCTTTTATTTATTAATAAAATATATGAAATTAAAATGTGATACAGATAAAAAGAATGGTACACAAAGTGATACAGAACATCTAAAATCCATATTTAGTATGTATGTTTTATCCATGTGTTAGACAAATAAAAAAAAAAATAATAATAATAAAAAAGGTGAGGGAGAGTAATTGAACATTTCTTGAAAATATTGAAAACAAAACTCTTTTTTGTAGGAGATTTCTCAGACTGATGTTTCAATCAAACTGCCTACCCGG |
| *pGO* | CGTAATGAATTTAGTGACGGATCGAAATTGAGGAGTCACTAAATTTTCTGTTTTTAGTGATTTGGTTGGAGATAGCCTAATAAGCAATCCAAATAGCAAATTATCGTTTCCTGTCTTTTTTGACTAAATCATGTTTTCCTCTGTCCACTCTATCTATAGGCTATAATAAATGCGTTAGAATGAAATTAAATCCCTTTCCATCTAAAAAAAATACAAAAAATCAATGTCACTGAGAATGAAATAAGAATCTTCATTTCAATGTTTTCATTTTGTTTGGTAGGGAAAATTTGAATACGATGGTTATTCTTTCTTTTTTTTTTTTTAGTCCCATTAATAGTAAACAGATTTTAAAATTCTTTCGAAAGTCTTTGTTCAACAAAAAAATCTTAACTGAGAAAAAAAATACTTGACTGAACAAACTAGCTCAGCTTGCAATCCACTGTTATGAGCAGAGAAACTGTGGTACATGGAAAAAACTTTTTTATTCCCCATAGAAATGACCAACTAGCTTTTAGATTGAGAAATCAGTAATATTAGTCTTTAAATTATTTTTTTAATTTTAATTTAATTCTTAATTTTTATTTTAATTTTTTAGTCTTTAAATAATTATTTCTAAAAATTTTAATCATTATCATTAATTTCATAATAAAATTTAACAATAAAAATTTGTGAGTCTCACAAAACTAATGAACAACGTTAAAAAATACATTTTACTTTATTCAACTCATCATTACTCTGTTAAACACAATAATGATGGTCTATTTGAATATTTGAAATATTTTTGAGGTTTATGTGAAATTTTATTTAAATACATAAAAATTTTCTCCAAGAAAATTTCCCTCTAAATTTTGAGAAATTTCTCTAATTAGATATAGGAAATATTTTTAGGGTTTAAACTTGTTTTCGGAATTTAGAGAAAAAATCACATAAACTCTAAGAATATTTCAGGTACTCAATTAGACATCATTGCTTTATTTAAAAGAGTAATGAGTAAGTGAAATAATTTTTTAATCATATTCATGATTCAAGTGAGATTCACATGACTTTTACTACTGATTTTTTTTATAAAATTAATGGCGAACATTAAAACTTTTTAAAAATAAATGTGCAGAACTAAAAAATGAAAACAAAATTTAAATATTAAGTTAAGATAAAAAATAATTTACACACTAATATTATATTATATTGCTTCTTAGAAAATTTTGAACGCATATCTTTTTGGTTTGGGGGTAAATTATGATTTCAGGGCAGCCATTTCAATATTAATAAGATAAAATAATGTAATGTATTATGTGCATTACACGTGTACTTATTAGTAGAATCATTTAACGTTTTTTCCTCCAAAAGAGAAAAAGGAATCATTACACGTAGCAGCAGCCCTTATATAAATATATGATTATTCTTTCGTCATTTACTCGCAACTAGTCAAAATAAGTGGACAATTATAATTTACAAAAAAGTTAACCTGAAATAGCAGCCAAATCTGAACTAAAACTAAAACAGCAATAAAATATTATGTATTCTCAGCCACCTTTGAGTCGCCCTATTATATATATATAATGAGTGGGATGCTAAGCTAAAGAAATCATTCCCCATTTTCCTTCCTTCCTTCCTTCTTCAGTCTCCTCTCTTGAACAAAACAC |
| *pT16H2* | TAGGTAGAGAAAGTTGACCAAAATCAAGATTTTTGCTGTGTAGTTTGCTTAACACTAGTAAGATGGAATGGCTTTTATAGATTTGAAGAAAGATACTTTCAGCTGTGTGGCTGTTGTTTATTTGCTTAAACATGGAATGGCTTTTATTGAGAAATAAACACCTTTATTTTCCACACGAGAATCATAGATTTCTCAAAAAGAGTAAAATTAGGGGGAAAAAAAAGAGAGAGAGAGAGAATTATAGTTTTTTCCTTCTATGGATATAAGTAGTTTTTCCATAAATAAACATTTTTATCCTATTCTAACACACAATTAGTTTGATTGATATTGATCCCCATAGAATTACTTGTATTTCTAGGCTCCCACCACTCTGGATCATCTCTACCATTTTTCTATACTATTCTTTATACTATTTCTTTTATAATATATATTTTTTTAATTTTTATCTTTTCCCTTAAGAATTTTTTTCTTTTGATAAATTTTACTGTTAATACAAAAGCAGTATCTTGAATTTTGTCATTATATCAATTAAGTATTCAATCTTTCAAAATAACAAGAGAATAATTTTTCTTTTCTAGTAAACTGCATTGAACGGAAACTGATCAGAAATGAGTAGTTTCTGTTCTACCCTAATAAAATTTGGAAAACCAACTTTTATCCTTGCATAGGAGGGGAGCTGGTCATCAAAAATATAATAAAAAACATTGTGATATTGTCAAAAAGAATAATCTGACAATGGACAAAGTTCATGGTAATTTTTTTGTATTAATCGTTCCCCAGAATATCTTTTTGATAATTTCATATATTTTAGAAGTTACAACTTTTTAATCTCTATAATATTTCTTACCCATTAAAGTTGATTGCAAGCAAAACAATGGTTTTTTAAGTCACAATAATGGTGTTATGAAACTTTTATATGCTTTTTGAGGATTTGGAGAAGGAAGTGAAAGCTTTGGTATATGGTTTTTAAGGGTCTGGGGAAGGAAGAGAAAGAAACTCATGCCATTTGTTTCTGCTGATAGGATTTGGTAGTAATTGGGTATTTTGATGGTCTTGGAAGAGAATTTTTTTTTAATTATATTTCTTCCCTCCATTTTGATGACAAGCTTTCCTCCTATAAAAGGATAAATTTAAGTTTTCCAAATTTTATTAGGGCATAACATACATTACTCATTTCTGATCAGTTTTCGTTCAATTTATTTTATCGAAAAAGCAAAATTATTCCTTAATTTTCTTTTTCAAAGATTAGGTGCTTAATTGACATAATGGACAAAGTTCAAGGTACTTTTTTTATATTAACCTTAAATTTTATTAATAAAATATATCAAATTAAAAATAGCATTACCATACCACCGCCCATCACATCAATGAGATTACT |
| *pSTR1* | GTTGACTGTAAATGATGGGAGTGAAGCAGAGAGGGGCAGCTATGGGGTATGGTTTGATACTTAGAGGGACAGTGAAGGAATATAATAAGCCCACAAACTCACCAAACAAGGTCCAAGGGAAATCACTTTCAAAGAAGGCGGTCTAAGAGTGATGTACCACGTGGAGTAATATTTATAGGCTTATCCAATATACTTATGCATTCTTTTATTCCTTTATTTTGTATATATCATAAATATATTTTTATCGGTTTATACATCAATTAATAATTATAATTCCTAATACTAATTATTGTAAAATAATTATATGATAAATAGCGATTTAATTACAAAATGTTTCGCTTGTCTAATTCGTATTTTCGACTATATATTAAAATCATAATACTTTTGCTAGATCTTTTTGTTTGTCCAATTTCTGTAATGAATAATATTATTGTTTTAATATGATAATAAATAAATTAAACTCGTAAAATGATATTATAGGAAGATTTTAAAATTTTTAGTAGCAGAATTATTTTTTTCAAATAGATTTTTTTTTGAAATCAGTTGTAGGGATTAATTAATTATGAAAATACAAACAATAAAGATC |
| *pT19H* | CCATGAGGTAGAAATTATGAAAGAAATTAAGGTAATAAATTGATTTTTCTTTTATACCTTTTCCTAATATTGCATGAATGGGTACCGGCTCTAAAAAATGGATGTAGTTTGTACATATTAATCTTAGCATATATTATATTTAATTATAGAGTATCGTCGGAAGGCGCTTCCAAACTGTAGGATCCGTAGGAGGAAGCCGTAGATGATGACGGACATATGGTCGATGCGTAAGATGAAGCGGCAGCATCGGAACGTGCCAGTAACTGGTGGCTGTGTTGGTGGATCTTGATCTGTACGTATCTATGGATCCTGCTATACTTGAGTCAATGAATTTTACTATGTCGTAGGCTGTGACTTCTACTGTGTCGAGGGCGTTAGATCCTTTCTCTTACGTCCTCGAGCCATCAAACCTTTAATATAAGTAAAAGGATAGTATTTGTTACGCCTCTAATTCACATACCCACACAAACTCAACTGCGTCTAATAGTCATTGGTAATTTATTTACCGACGGCGTTCATCATCGGTAAAATGTCATCACTTTCTGGCACACCCTTTCCTTCTTTTTTTAGTTGGAAATGTGAAAATCCATTGACATACAAGTTTTCATCGGTACCCCATCAGTGTTTAGTAACGATAAACTGTAATGTTAGTAAAGTCCGTCGGTAACTGTTTATTTATATATATATATATATATATAATATGCATAATTGATCACGCGGCTTTATATGAATGGACGATCCGTTTTCTCCGCTATGTGTTCGTTGGGCAATCTTCTTCTTAGCATGCCTTTCATTGACTCATTGGTGTTTCTTTAGCTATTAAATTAGTATTAAATTAAACTATTCCAATGCTTTAAAATGAAATGACATAAAAAAATCAGTATAATAATACATCAATGATCATGTAAATCTGGACTTTTCTGAACAAATCCATCTATAAATGATGAGAATATCTAAAGAAGAGAGCCATTCATAGCAAAAACAACTAGATTTCTCTAGCAAGAAAAAT |
| *pG10H* | ATCAAACCCTTGCTCTTCCTTATATATAGAGTTTTTTTTTTTTTACAAATTTATGGACAATGAATTTTTACAAGGATGAATTTCTATTAATCACTTTTAGATTTATAATTTTATAAGTTTTTTTCCACCATATCATTTTTTACTTCCTTTATCATAATTAATTGGGTTCTTTTTCTAATTAATAGCATCATGACTGTCATATATATATATATATATATATATAGAGAAAGTTAAGCTCTAAACCCTAGCAAAAAGAAAATAAAATATATTGAGAAGGCAGAATTGTGCAAGGAAGCAAAGGAGAAGAAAAAAATAGCAAACTAGATACATGTGCGAAAGATTAATTAAAGGAATTTGCATTGTATCAATTGTTTGATGAATATTTTTTCTTAAAATTTGCTATTTAACATTTTCCTTGTACTATTTGATAAAAAAAAATTTTTATTTAGTAATTGTATTAAGTTTTCTCGTTATAGAATTTCTCGCTTCAAGGATCAAGAGGAAAGTTTTAATATTTAATTTATCTATCCAAATAAAAATTCAAATAATTTTTTGTTTTTTTATTTTGGCTTTTTATTATTTTATGGTTGCAATTTTTTTTATTAAATTAATTGATTTTTGTTAATTTTCTTTTACATTGTTGTCAAATTATAATTATCTATTCGTATGTAATTTATTGGATGATGTTATAATTCTAAAAACTTTTAATTATGAAAATATTTCATTAAATTTTAATATTTAATTTAAATAATAATATTCATCTATTTAATGACCTCACGTGGGTTGATTGGTTAGTAATATAAAAAGTAAATAAATATTTTTAAAAAAAATTAGACAAGTAACATAAAATATTTTAAGTAACTCGAGCTTCTCGAGTTCTGTCCGACTTGGCCAGTAGCTATCAATCTCCATTCTCCAGTAGTAGTATATTAAAGAACACTGCCTCAAATCTCCATAGCCACAGAGCAAGCAAGGAGAACAAATTGTACACTTCCATTCC |

**Table S5. Oligonucleotides used to amplify and clone VIGS fragments targeting ZCTs in various configurations.**

| **Simultaneous targeting of *ZCT1* through *ZCT6*** | | |
| --- | --- | --- |
| **GOI** | **Oligonucleotide Name** | **Sequence (5' to 3')** |
| *ZCT1 and ZCT2* | KF235_VIGS_frag_ZCT1-2_F | tttggtctcaTATGTATGAGTGCAAAACTTGTAAAAAGC |
|  | KF236_VIGS_frag_ZCT1-2_R | tttggtctcaCGTCTCATATGACCTCCGA |
| *ZCT3* | KF237_VIGS_frag_ZCT3_F | tttggtctcaGACGCAACAACAACCCACCGAAGA |
|  | KF238_VIGS_frag_ZCT3_R | tttggtctcaAGTGCTTGGTAAGAAGAAAAGG |
| *ZCT5* | KF239_VIGS_frag_ZCT5_F | tttggtctcaCACTAGACATAGAAATATTTCTACAATTACAAAATCTA |
|  | KF240_VIGS_frag_ZCT5_R | tttggtctcaACCAAAGCAGGGGCTG |
| *ZCT6* | KF241_VIGS_frag_ZCT6a_F | tttggtctcaTGGTATGGCTCTTGAAGCCTTGA |
|  | KF242_VIGS_frag_ZCT6a_R | tttggtctcaACCGATACAGAATCATCGC |
| *ZCT4* | KF243_VIGS_frag_ZCT4_F | tttggtctcaCGGTTACTGAAAAGAAGCAGAGAAGATC |
|  | KF244_VIGS_frag_ZCT4_R | tttggtctcaCACCTCTGTGTCCTCCTAATGCTTG |
|  |  |  |
| **Additional Oligos for Simultaneous targeting of *ZCT1, ZCT2,* and *ZCT3* or *ZCT4, ZCT5,* and *ZCT6*** | | |
| **GOI** | **Oligonucleotide Name** | **Sequence (5' to 3')** |
| *ZCT3* | KF246_VIGS_frag_ZCT3_last_position_R | tttggtctcaCACCAGTGCTTGGTAAGAAGAAAAGG |
| *ZCT5* | KF247_VIGS_frag_ZCT5_first_position_F | tttggtctcaTATGAGACATAGAAATATTTCTACAATTACAAAAT |
|  |  |  |
| **Oligos for Targeting single *ZCTs* with multiple Fragments** | | |
| **GOI** | **Oligonucleotide Name** | **Sequence (5' to 3')** |
| *ZCT1 and ZCT2* | KF295_VIGS_frag_v2_ZCT1-2_300 bp_second and last position_F | tttggtctcaGACGCAATCCGCAAATTGTT |
|  | KF296_VIGS_frag_v2_ZCT1-2_300 bp_second and last position_R | tttggtctcaCACCTCTCATATGACCTCCGAGAG |
| ZCT3 | KF297_VIGS_frag_v2_ZCT3_300bp_first position_F | tttggtctcaTATGAGATCCTCCTAAGCTTCTCTAC |
|  | KF298_VIGS_frag_v2_ZCT3_300bp_first position_R | tttggtctcaCCGCTAGCCGCGTTTC |
|  | KF299_VIGS_frag_v2_ZCT3_200bp_second and last position_F | tttggtctcaGCGGCAACAACAACCCACCGAA |

**Table S6. VIGS fragment sequences assembled into pTRV2 plasmids**

| **pKF317 - pTRV2-ZCT1_2-ZCT3-ZCT5-ZCT6a-ZCT4** | |
| --- | --- |
| **Target(s)** | **VIGS sequence** |
| ZCT1/2,ZCT3, ZCT5,ZCT6, ZCT4 assembled | TATGAGTGCAAAACTTGTAAAAAGCAATTTCTCACTTTTCAGGCTTTAGGCGGTCACCGAGCGAGTCACAAGAAGCTTAGATTAATGGCGGCAGATCTACTCCATCAATCTTTAGCGGTGACGAAGCCGAAAACTCATGCTTGTTCTATATGTGGCCTCGAATTTCCATTAGGTCAGGCTCTCGGAGGTCATATGAGACGCAACAACAACCCACCGAAGAAGAATACCTCGCTCTCTGTCTTATTATGCTTGCTCGCGGCGGAGCTCCATCAACTACTACTCTTCCTCTTCCACCTCCGCCGCAGCAACACAATATTCCTTCTTCTTCTTCTTCAGATCCTCCTAAGCTTCTCTACAAGTGTTCCGTTTGTGATAAGGCCTTTTCTTCTTACCAAGCACTAGACATAGAAATATTTCTACAATTACAAAATCTATTGAATCATCGGAGAAGCTGCCGCCACCGTCACCGCCGGTTAGAAGTATTTTTCCATTGGATCTTAATTTGCCGGCACCGGCGGAAGATGATCATGGCGGCAGGGAAATGAAGTTTCAGTTTTCAGCCAATAAACAGCGTCTTGTATTCTCAGCCCCTGCTTTGGTATGGCTCTTGAAGCCTTGAATTCTCCAGCGGCAGCAGCTCCTCGATTTCCAGACGCTACCGACGTTAATACTTTCGATTCTTTGACGAAAAGAAAGCGTTCAAAACGTCCTCGTTCTGAAACTCCGCCGACTGAAGAAGAATATTTGGCTGCCTGTCTCGTCATGCTCGCTCGCGGTCGCGGCGATGATTCTGTATCGGTTACTGAAAAGAAGCAGAGAAGATCACGATAGGGAGGCCATTGAATCCTTAGCCATGGCAAATTGTTTGATGCTTTTATCAACTGTGGGCAAAACTACTAATAATTCTTCTCCTTTAATAGGATCAGATAATGATCGAGTTTTCTCGTGCAAAACATGTAACAAAAAGTTTCCGACATTTCAAGCATTAGGAGGACACAGA |
| ZCT1/2 | TATGAGTGCAAAACTTGTAAAAAGCAATTTCTCACTTTTCAGGCTTTAGGCGGTCACCGAGCGAGTCACAAGAAGCTTAGATTAATGGCGGCAGATCTACTCCATCAATCTTTAGCGGTGACGAAGCCGAAAACTCATGCTTGTTCTATATGTGGCCTCGAATTTCCATTAGGTCAGGCTCTCGGAGGTCATATGA |
| ZCT3 | CAACAACAACCCACCGAAGAAGAATACCTCGCTCTCTGTCTTATTATGCTTGCTCGCGGCGGAGCTCCATCAACTACTACTCTTCCTCTTCCACCTCCGCCGCAGCAACACAATATTCCTTCTTCTTCTTCTTCAGATCCTCCTAAGCTTCTCTACAAGTGTTCCGTTTGTGATAAGGCCTTTTCTTCTTACCAAG |
| ZCT5 | AGACATAGAAATATTTCTACAATTACAAAATCTATTGAATCATCGGAGAAGCTGCCGCCACCGTCACCGCCGGTTAGAAGTATTTTTCCATTGGATCTTAATTTGCCGGCACCGGCGGAAGATGATCATGGCGGCAGGGAAATGAAGTTTCAGTTTTCAGCCAATAAACAGCGTCTTGTATTCTCAGCCCCTGCTT |
| ZCT6 | ATGGCTCTTGAAGCCTTGAATTCTCCAGCGGCAGCAGCTCCTCGATTTCCAGACGCTACCGACGTTAATACTTTCGATTCTTTGACGAAAAGAAAGCGTTCAAAACGTCCTCGTTCTGAAACTCCGCCGACTGAAGAAGAATATTTGGCTGCCTGTCTCGTCATGCTCGCTCGCGGTCGCGGCGATGATTCTGTAT |
| ZCT4 | TACTGAAAAGAAGCAGAGAAGATCACGATAGGGAGGCCATTGAATCCTTAGCCATGGCAAATTGTTTGATGCTTTTATCAACTGTGGGCAAAACTACTAATAATTCTTCTCCTTTAATAGGATCAGATAATGATCGAGTTTTCTCGTGCAAAACATGTAACAAAAAGTTTCCGACATTTCAAGCATTAGGAGGACACAGA |
|  |  |
| **pKF329 - pTRV2-ZCT1_2-ZCT3** | |
| **Target(s)** | **VIGS sequence** |
| ZCT1/2,3 assembled | TATGAGTGCAAAACTTGTAAAAAGCAATTTCTCACTTTTCAGGCTTTAGGCGGTCACCGAGCGAGTCACAAGAAGCTTAGATTAATGGCGGCAGATCTACTCCATCAATCTTTAGCGGTGACGAAGCCGAAAACTCATGCTTGTTCTATATGTGGCCTCGAATTTCCATTAGGTCAGGCTCTCGGAGGTCATATGAGACGCAACAACAACCCACCGAAGAAGAATACCTCGCTCTCTGTCTTATTATGCTTGCTCGCGGCGGAGCTCCATCAACTACTACTCTTCCTCTTCCACCTCCGCCGCAGCAACACAATATTCCTTCTTCTTCTTCTTCAGATCCTCCTAAGCTTCTCTACAAGTGTTCCGTTTGTGATAAGGCCTTTTCTTCTTACCAAGCACT |
| ZCT1/2 | TATGAGTGCAAAACTTGTAAAAAGCAATTTCTCACTTTTCAGGCTTTAGGCGGTCACCGAGCGAGTCACAAGAAGCTTAGATTAATGGCGGCAGATCTACTCCATCAATCTTTAGCGGTGACGAAGCCGAAAACTCATGCTTGTTCTATATGTGGCCTCGAATTTCCATTAGGTCAGGCTCTCGGAGGTCATATGA |
| ZCT3 | CAACAACAACCCACCGAAGAAGAATACCTCGCTCTCTGTCTTATTATGCTTGCTCGCGGCGGAGCTCCATCAACTACTACTCTTCCTCTTCCACCTCCGCCGCAGCAACACAATATTCCTTCTTCTTCTTCTTCAGATCCTCCTAAGCTTCTCTACAAGTGTTCCGTTTGTGATAAGGCCTTTTCTTCTTACCAAG |
|  |  |
| **pKF330 - pTRV2-ZCT5-ZCT6a-ZCT4** | |
| **Target(s)** | **VIGS sequence** |
| ZCT5,6,4 assembled | AGACATAGAAATATTTCTACAATTACAAAATCTATTGAATCATCGGAGAAGCTGCCGCCACCGTCACCGCCGGTTAGAAGTATTTTTCCATTGGATCTTAATTTGCCGGCACCGGCGGAAGATGATCATGGCGGCAGGGAAATGAAGTTTCAGTTTTCAGCCAATAAACAGCGTCTTGTATTCTCAGCCCCTGCTTTGGTATGGCTCTTGAAGCCTTGAATTCTCCAGCGGCAGCAGCTCCTCGATTTCCAGACGCTACCGACGTTAATACTTTCGATTCTTTGACGAAAAGAAAGCGTTCAAAACGTCCTCGTTCTGAAACTCCGCCGACTGAAGAAGAATATTTGGCTGCCTGTCTCGTCATGCTCGCTCGCGGTCGCGGCGATGATTCTGTATCGGTTACTGAAAAGAAGCAGAGAAGATCACGATAGGGAGGCCATTGAATCCTTAGCCATGGCAAATTGTTTGATGCTTTTATCAACTGTGGGCAAAACTACTAATAATTCTTCTCCTTTAATAGGATCAGATAATGATCGAGTTTTCTCGTGCAAAACATGTAACAAAAAGTTTCCGACATTTCAAGCATTAGGAGGACACAGA |
| ZCT5 | AGACATAGAAATATTTCTACAATTACAAAATCTATTGAATCATCGGAGAAGCTGCCGCCACCGTCACCGCCGGTTAGAAGTATTTTTCCATTGGATCTTAATTTGCCGGCACCGGCGGAAGATGATCATGGCGGCAGGGAAATGAAGTTTCAGTTTTCAGCCAATAAACAGCGTCTTGTATTCTCAGCCCCTGCTT |
| ZCT6 | ATGGCTCTTGAAGCCTTGAATTCTCCAGCGGCAGCAGCTCCTCGATTTCCAGACGCTACCGACGTTAATACTTTCGATTCTTTGACGAAAAGAAAGCGTTCAAAACGTCCTCGTTCTGAAACTCCGCCGACTGAAGAAGAATATTTGGCTGCCTGTCTCGTCATGCTCGCTCGCGGTCGCGGCGATGATTCTGTAT |
| ZCT4 | TACTGAAAAGAAGCAGAGAAGATCACGATAGGGAGGCCATTGAATCCTTAGCCATGGCAAATTGTTTGATGCTTTTATCAACTGTGGGCAAAACTACTAATAATTCTTCTCCTTTAATAGGATCAGATAATGATCGAGTTTTCTCGTGCAAAACATGTAACAAAAAGTTTCCGACATTTCAAGCATTAGGAGGACACAGA |
|  |  |
| **pKF398 - pTRV2-ZCT1/2 (200 bp)-ZCT1/2 (300 bp)** | |
| **Target(s)** | **VIGS sequence** |
| ZCT1/2 assembled | TATGTATGAGTGCAAAACTTGTAAAAAGCAATTTCTCACTTTTCAGGCTTTAGGCGGTCACCGAGCGAGTCACAAGAAGCTTAGATTAATGGCGGCAGATCTACTCCATCAATCTTTAGCGGTGACGAAGCCGAAAACTCATGCTTGTTCTATATGTGGCCTCGAATTTCCATTAGGTCAGGCTCTCGGAGGTCATATGAGACGCAATCCGCAAATTGTTTGATGCTTCTTTCTAAAGTCGGATTATTATCAGAAATGGAGAAATCAGCAGTACCGGCGTTGAAACCCGGCGCCGGCGGACGAATTTATGAGTGCAAAACTTGTAAAAAGCAATTTCTCACTTTTCAGGCTTTAGGCGGTCACCGAGCGAGTCACAAGAAGCTTAGATTAATGGCGGCAGATCTACTCCATCAATCTTTAGCGGTGACGAAGCCGAAAACTCATGCTTGTTCTATATGTGGCCTCGAATTTCCATTAGGTCAGGCTCTCGGAGGTCATATGAGA |
| ZCT1/2 (200 bp) | TATGAGTGCAAAACTTGTAAAAAGCAATTTCTCACTTTTCAGGCTTTAGGCGGTCACCGAGCGAGTCACAAGAAGCTTAGATTAATGGCGGCAGATCTACTCCATCAATCTTTAGCGGTGACGAAGCCGAAAACTCATGCTTGTTCTATATGTGGCCTCGAATTTCCATTAGGTCAGGCTCTCGGAGGTCATATGA |
| ZCT1/2 (300 bp) | CAATCCGCAAATTGTTTGATGCTTCTTTCTAAAGTCGGATTATTATCAGAAATGGAGAAATCAGCAGTACCGGCGTTGAAACCCGGCGCCGGCGGACGAATTTATGAGTGCAAAACTTGTAAAAAGCAATTTCTCACTTTTCAGGCTTTAGGCGGTCACCGAGCGAGTCACAAGAAGCTTAGATTAATGGCGGCAGATCTACTCCATCAATCTTTAGCGGTGACGAAGCCGAAAACTCATGCTTGTTCTATATGTGGCCTCGAATTTCCATTAGGTCAGGCTCTCGGAGGTCATATGAGA |
|  |  |
| **pKF399 - pTRV2-ZCT3 (300 bp)-ZCT3 (200 bp)** | |
| **Target(s)** | **VIGS sequence** |
| ZCT3 assembled | AGATCCTCCTAAGCTTCTCTACAAGTGTTCCGTTTGTGATAAGGCCTTTTCTTCTTACCAAGCACTCGGAGGACATAAGGCAAGTCACAGGAAACTCTCCACAGCCTCCGCCGGAGGTGGAGATGACCAGTCAACGACTTCAACTTCCACAACTACCGCTGCCGCCGCTACCGGAAGGACTCACGAGTGTTCCATTTGTCATAAGTGTTTTCCTTCTGGACAAGCTTTGGGAGGACACAAGCGTTGCCACTACGAAGGCGGCGCCGGCGCCGTCGGAAGTACCGGAAACGCGGCTAGCGGCAACAACAACCCACCGAAGAAGAATACCTCGCTCTCTGTCTTATTATGCTTGCTCGCGGCGGAGCTCCATCAACTACTACTCTTCCTCTTCCACCTCCGCCGCAGCAACACAATATTCCTTCTTCTTCTTCTTCAGATCCTCCTAAGCTTCTCTACAAGTGTTCCGTTTGTGATAAGGCCTTTTCTTCTTACCAAGCACT |
| ZCT3 (300 bp) | AGATCCTCCTAAGCTTCTCTACAAGTGTTCCGTTTGTGATAAGGCCTTTTCTTCTTACCAAGCACTCGGAGGACATAAGGCAAGTCACAGGAAACTCTCCACAGCCTCCGCCGGAGGTGGAGATGACCAGTCAACGACTTCAACTTCCACAACTACCGCTGCCGCCGCTACCGGAAGGACTCACGAGTGTTCCATTTGTCATAAGTGTTTTCCTTCTGGACAAGCTTTGGGAGGACACAAGCGTTGCCACTACGAAGGCGGCGCCGGCGCCGTCGGAAGTACCGGAAACGCGGCTA |
| ZCT3 (200 bp) | CAACAACAACCCACCGAAGAAGAATACCTCGCTCTCTGTCTTATTATGCTTGCTCGCGGCGGAGCTCCATCAACTACTACTCTTCCTCTTCCACCTCCGCCGCAGCAACACAATATTCCTTCTTCTTCTTCTTCAGATCCTCCTAAGCTTCTCTACAAGTGTTCCGTTTGTGATAAGGCCTTTTCTTCTTACCAAGCACT |

**Table S7****.** **RT-qPCR oligonucleotide sequences**

| **GOI** | **Oligonucleotide Name** | **Sequence (5' to 3')** |
| --- | --- | --- |
| ZCT1 | Zct1-F | AATCTTTAGCGGTGACGAAGCCGA |
|  | Zct1-R | CGTTGTCCTCAGGCGTCAAATTCA |
| ZCT2 | Zct2-forward | TTTCCATCGTTTCAAGCTCTCGGC |
|  | Zct2-reverse | ATTACCGGACGCCGAATCACTCAT |
| ZCT3 | Zct3-forward | CAGCAACAACAACCGACCGAAGAA |
|  | Zct3-reverse | TTGCCTTATGTCCTCCGAGTGCTT |
| ZCT4 | KF062_ZCT4_qF | GGACACAGAGCAAGTCACAA |
|  | KF063_ZCT4_qR | CGCCAAGAGCTTGTCCTAAA |
| ZCT5 | KF064_ZCT5_qF | CACCGCCGGTTAGAAGTATT |
|  | KF065_ZCT5_qR | GACGCTGTTTATTGGCTGAAA |
| ZCT6 | KF066_ZCT6a_qF | GCCACTACGAAGGACACATAG |
|  | KF067_ZCT6a_qR | CATGAGGCTTGTGACTAGAGATAC |

**Table S8. Top 5 non-redundant Arabidopsis Cys2-His2 zinc finger proteins homologous to the C. roseus ZCT proteins**
